# Handgrip strength and the risk of major depressive disorder: a two-sample Mendelian randomisation study

**DOI:** 10.1136/gpsych-2022-100807

**Published:** 2022-09-27

**Authors:** Nanxi Li, Rui Zhou, Bin Zhang

**Affiliations:** 1 Psychiatric and Psychological Neuroimage Lab (PsyNI Lab), Affiliated Brain Hospital of Guangzhou Medical University, Guangzhou, Guangdong, China; 2 Institute of Mental Health, Tianjin Anding Hospital, Tianjin Medical University, Tianjin, China

**Keywords:** depression

## Abstract

**Background:**

Major depressive disorder (MDD) is a common psychiatric disease and a leading cause of disability worldwide. Handgrip strength (HGS) as an objective physical fitness test is a practical index for identifying many diseases. Previous studies drew different conclusions about the relationship between HGS and MDD.

**Aims:**

We aim to explore whether HGS has an effect on the risk of MDD.

**Methods:**

HGS-related single-nucleotide polymorphisms identified by a genome-wide association study were used as instrumental variables in this Mendelian randomisation (MR) study. Summary data on MDD were obtained from the Psychiatric Genomics Consortium. Four methods were applied, including inverse variance weighted (IVW), MR Egger, weighted median and weighted mode. Additional sensitivity analyses, including leave-one-out, heterogeneity test, pleiotropy test and confounders identification, were conducted to test the robustness of our results.

**Results:**

Each 1 kg increase in left HGS is associated with a 21.95% reduction in the risk of MDD (OR_IVW_ = 0.781, 95% CI: 0.650 to 0.937, p=0.009), while no significant correlation exists in the estimation of right HGS (p=0.146). Sensitivity analyses demonstrated statistical significance (β_IVW_ = −0.195, p=0.023) after excluding some genetic loci that cause pleiotropy.

**Conclusions:**

Increased left HGS is associated with a reduced risk of MDD. In the future, it may be used as an index for the clinical screening, observation and treatment of MDD.

**WHAT IS ALREADY KNOWN ON THIS TOPIC**

- It is necessary to find a suitable biomarker that can be an early predictor of the risk of major depressive disorder.

**WHAT THIS STUDY ADDS**

- This work shows that increased handgrip strength is likely to indicate a lower risk of major depressive disorder.

**HOW THIS STUDY MIGHT AFFECT RESEARCH, PRACTICE OR POLICY**

- According to current outcomes, handgrip strength would be expected to become an indicator with suggestive significance for the risk of major depressive disorder.

## Introduction

Major depressive disorder (MDD) is a common psychiatric condition that represents a leading cause of disability worldwide and is associated with deficits in cognition, including executive function and memory.^1^ These cognitive deficits impair social and occupational functioning, contributing to the personal, social and economic burden associated with mood disorders.^2^ However, preventing MDD has been challenging, with few established protective factors, particularly modifiable targets for prevention. Recent years witnessed the increasing recognition of the importance of objective physical fitness tests as indicators of physical, mental and cognitive outcomes in the general population.^3^ Handgrip strength (HGS) is quickly assessed and provides an objective measure of muscular fitness, emerging as an important biomarker of overall health and disease status. Assessing HGS asymmetry may provide insights into HGS, as one study has verified that handgrip asymmetry would hasten mortality.^4^ Owing to the strength of the evidence in this area, HGS is now considered an easily administered and clinically useful index of cognitive decline across the lifespan.^3^

Despite growing epidemiological evidence regarding the relationship between HGS and mental illness, studies using HGS or other objective physical performance measures to explore strength loss as an indicator of MDD and other common mental health conditions have yielded inconsistent results.^5 6^ Moreover, different dominant hands lead to different health outcomes; for example, different times to mortality were found in populations with a different dominant hand.^4^ Few studies have investigated lifestyle factors concerning MDD risk among people with normal HGS. Yet these results could be possibly biased by factors that influence both HGS and MDD. Consequently, findings of pre-existing observational studies are insufficient to draw a definitive conclusion on the causal relationship between HGS and MDD due to their susceptibility to reverse causality and potential confounders.

Mendelian randomisation (MR) analysis, in which genetic variants are used as instrumental variables (IVs), is a relatively precise epidemiological approach to evaluate the causal relationship between exposure and outcome with less susceptibility to potential confounding factors and reverse causality. Since genetic variations strictly adhere to the principle of random distribution at conception, they are generally independent of environmental risk factors, antecedent risk factors and disease development. One MR study has demonstrated that genetically predicted MDD was causally associated with an increasing risk of HGS decline.^7^ However, to date, there has been no report of the causality from HGS to MDD.

The aim of this study was to use population-scale data from large-scale genome-wide association studies (GWASs) to establish how muscular function measured by HGS relates to MDD and assess the impact of potential confounders on the outcome using a two-sample MR method. The findings, in turn, will improve our current understanding of the use of the handgrip dynamometer as an indicator of overall cognitive status, along with providing new insights into the contributing factors and possible interventions for cognitive functioning in people with and without psychiatric conditions.

## Materials and methods

GWAS data of 634 094 subjects were included in this study. All selected data sets have obtained ethical approval.

### Data sources of HGS and MDD

Depression severity of 59 851 patients with MDD and 113 154 healthy controls from the Psychiatric Genomics Consortium (https://www.med.unc.edu/pgc/) was considered as the outcome.^8^ A GWAS dataset of 461 089 individuals from the Medical Research Council-Integrative Epidemiology Unit (MRC-IEU) (http://www.bristol.ac.uk/integrative-epidemiology/) based on the UK Biobank^9^ was selected as the exposed group in our study. These data are available on request to approved researchers.

**Figure 1:**
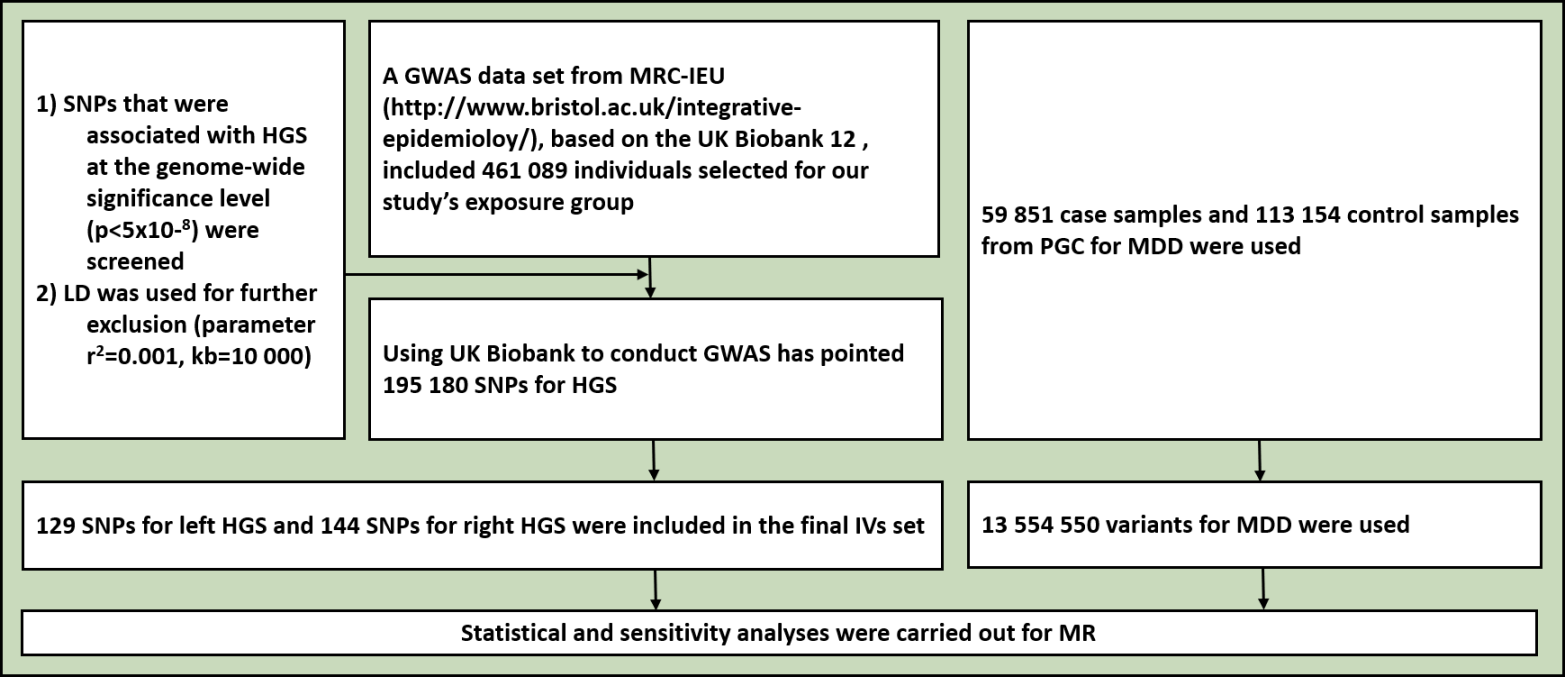
Flowchart for the enrolment. GWAS, genome-wide association study; HGS, handgrip strength; IVs, instrumental variables; LD, linkage disequilibrium; MDD, major depressive disorder; MR, Mendelian randomisation; MRC-IEU, Medical Research Council-Integrative Epidemiology Unit; PGC, Psychiatric Genomics Consortium; SNPs, single-nucleotide polymorphisms.

A previous GWAS based on the UK Biobank found that single-nucleotide polymorphisms (SNPs) explained approximately 23.9% of the variation in HGS across individuals.^10^ We screened out SNPs that were associated with maximal HGS at the genome-wide significance level (p<5×10^−8^) in the exposed group and used linkage disequilibrium (LD) for further exclusion (r^2^=0.001, kb=10 000). Finally, 129 SNPs associated with left HGS and 144 SNPs with right HGS were included in the instrument variants (IVs) set (figure 1, online supplemental table S1).

10.1136/gpsych-2022-100807.supp1 Supplementary data

### Statistical analysis for MR

MR analyses were conducted with the ‘Two Sample MR’ package (V.0.5.6) in R studio (V.4.0.3). This package harmonises exposed and outcome datasets containing information on SNPs, effect alleles, other alleles, effect sizes (odds ratios (ORs) converted to beta statistics by log transformation), SEs, p values and effect allele frequencies for the selected exposure instruments. MR analysis was conducted using inverse-variance weighted (IVW), MR Egger, weighted median and weighted mode.

### Sensitivity analysis for MR

Notably, in order to have a valid interpretation for the MR analysis, it is necessary to hold the following assumptions^11^ (figure 1):

1. The IVs are robustly correlated with HGS.
2. The IVs influence MDD only through their effect on HGS.
3. The IVs are independent of any confounders of HGS-MDD relation.

Sensitivity analyses examining whether these assumptions were satisfied were performed to confirm the robustness of our results. We conducted leave-one-out method, heterogeneity test and MR-Egger regression for horizontal pleiotropy^12^ and explored the potential confounders. These tests vary in principle but essentially capture the extent to which the effect for one or more instrument SNPs is exaggerated in magnitude, as would be the case if that SNP not only acted through the hypothesised pathway but through other unaccounted causal pathways.

The leave-one-out method was implemented to investigate whether the estimation of IVW analysis could be biased or determined by a particular SNP. It was performed based on the re-run IVW outcomes of the remaining SNPs after successively omitting one SNP each time. When the result is not affected by a specific SNP, the regression coefficients remain relatively stable.

The heterogeneity test was similar to that in the meta-analysis. Cochran’s Q test was used to examine the heterogeneity (p<0.05 was considered statistically significant).

In the MR-Egger regression, p<0.05 indicates the presence of horizontal pleiotropy. In addition, we used MR–Pleiotropy Residual Sum and Outlier (PRESSO) for further analysis.

To verify the third assumption, we employed additional MR analysis to investigate whether genetic predisposition towards HGS is associated with the potential confounders underlying the affecting factors of HGS. For instance, more alcohol consumption is related to both reduced HGS^13^ and increased risk of MDD.^14^ Larger waist circumference was frequently found in patients with MDD, compared with the general population.^15^ In addition, an independent negative association was found between HGS and metabolism-related markers, including waist circumference, triglycerides, high-density lipoprotein cholesterol (HDL-C), glucose and blood pressure (BP).^16^ Higher levels of triglycerides and lower levels of HDL-C were observed in case–control studies and meta-analyses.^17^ Glucose metabolism is an important pathway and mechanism for brain activities associated with MDD.^18^ Genes associated with blood pressure have genetic overlap with MDD.^19^ Overall, metabolism-related markers are considered potential confounders of the HGS–MDD relationship. Also, exercise and frailty are important confounders which are associated with HGS and MDD.^20 21^

Conventional MR was applied to investigate whether genetically predicted HGS was associated with these confounding factors or mediators. Data on alcohol assumption were obtained from the GWAS and Sequencing Consortium of Alcohol and Nicotine use, while genetic summary data on waist circumference were extracted from the Genetic Investigation of ANthropometric Traits (GIANT) (http://giant.princeton.edu/). Genetic instruments of triglycerides and HDL-C were obtained from 21 545 individuals in 14 cohorts of Europeans and the UK Household Longitudinal Study (http://understandingsociety.ac.uk). In addition, a data set of glucose was chosen from the published GWAS in the Meta-Analysis of Glucose and Insulin related traits Consortium (MAGIC) (https://magicinvestigators.org/), and information about blood pressure came from the International Consortium of Blood Pressure (ICBP). Data on exercise and frailty were obtained respectively from 350 492 Europeans recruited by Klimentidis *et al* and 175 226 Europeans by Atkins *et al*. (table 1).

**Table 1:** Extra Mendelian randomisation analysis to explore confounders of the HGS–MDD relationship

Confounder	First author	Consortium	Population	Sample size	Year	Website
Alcohol drinks per day	Liu M	GWAS and Sequencing Consortium of Alcohol and Nicotine use	European	335 394	2019	NA
Waist circumference	Shungin D	GIANT	European	127 997	2015	http://giant.princeton.edu/
Triglycerides	Kettunen J	NA (14 cohorts)	European	21 545	2016	NA
HDL-C	Prins BP	UKHLS	European	9796	2017	www.understandingsociety.ac.uk
Glucose	Manning AK	MAGIC	European	58 074	2012	https://magicinvestigators.org/
DBP	Evangelou E	ICBP	European	757 601	2018	NA
SBP	Evangelou E	ICBP	European	757 601	2018	NA
Exercise	Klimentidis YC	NA	European	350 492	2018	NA
Frailty	Atkins JL	NA	European	175 226	2021	NA

DBP, diastolic blood pressure; GIANT, Genetic Investigation of ANthropometric Traits; GWAS, genome-wide association study; HDL-C, high-density lipoprotein cholesterol; HGS, handgrip strength; ICBP, the International Consortium of Blood Pressure; MAGIC, the Meta-Analysis of Glucose and Insulin related traits Consortium; MDD, major depressive disorder; NA, not available; SBP, systolic blood pressure; UKHLS, the UK Household Longitudinal Study.

## Results

### Causal effect between HGS and MDD

One-kilogram increase in left HGS was associated with a 21.95% reduction of risk for MDD (OR_IVW_=0.781, 95% CI: 0.650 to 0.937, p=0.009). No significant correlation was found in the estimation of right HGS (p=0.146). In addition, MR Egger showed OR_left handgrip_ = 0.997 (p=0.986), OR_right handgrip_ = 0.984 (p=0.962). Weighted median showed OR _left handgrip_ = 0.783 (p=0.032), OR_right handgrip_ = 0.797 (p=0.038). Weighted mode showed OR _left handgrip_ = 0.770 (p=0.434), OR_right handgrip_ = 1.488 (p=0.130).

**Figure 2:**
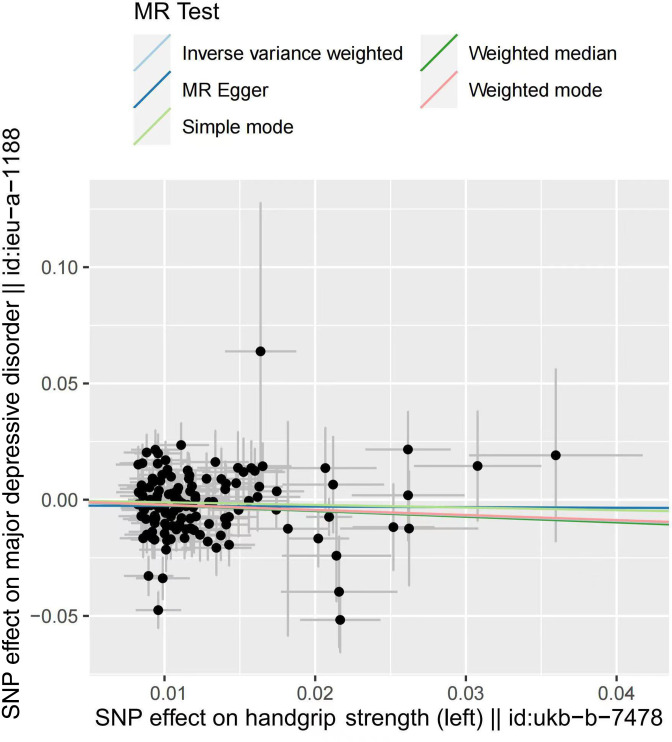
Scatter plot of the effect of left handgrip strength on major depressive disorder. MR, Mendelian randomisation; SNP, single-nucleotide polymorphism.

**Figure 3:**
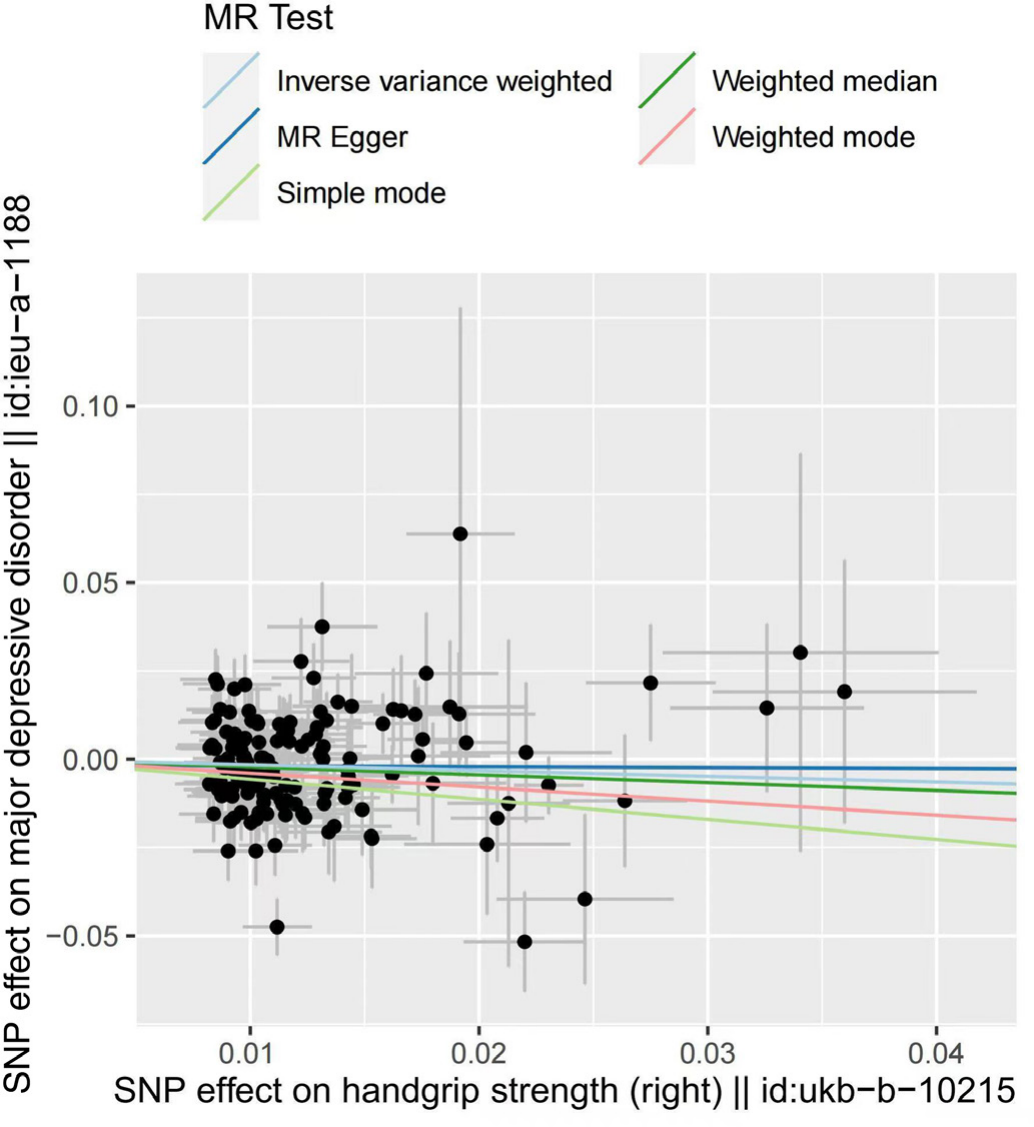
Scatter plot of the effect of right handgrip strength on major depressive disorder. MR, Mendelian randomisation; SNP, single-nucleotide polymorphism.

We recalculated the effect after excluding the overlapping SNPs between left and right handgrip, and the result again suggested that only the change of the left handgrip has an effect on MDD (left: OR_IVW after elimination_ = 0.813, p=0.049; right: OR_IVW after elimination_ = 0.915, p=0.386) (figures 2 and 3, online supplemental figures S1 and S2). Note that eliminating the overlaps may bias the result due to the potential violation of the first assumption of Mendelian randomisation study.

10.1136/gpsych-2022-100807.supp2 Supplementary data

### Sensitivity analysis for the assessment of three MR assumptions and MR analysis

The first assumption was satisfied since all screening SNPs were associated with HGS at the genome-wide significance level (p<5×10^−8^), and the F-statistic was 1122 for left HGS and 1005 for right HGS (F>100) (online supplemental table S2). Furthermore, we assessed if the correlation between genetically predicted HGS and MDD was impacted by the aforementioned potential confounders, that is, alcohol drinks per day, waist circumference, triglycerides, HDL-C, glucose, BP, exercise and frailty. Results showed that, except for frailty (OR_IVW right handgrip_= 0.871, 95% CI: 0.779 to 0.974, p=0.016; OR_IVW left handgrip_= 0.882, 95% CI: 0.796 to 0.977, p=0.016), no significant correlation was found between genetically predicted HGS and potential confounders (table 2). On the genome-wide level, our genetic instrument of HGS-associated SNPs was not significantly associated with any other phenotype momentarily. In addition, the leave-one-out method showed no abnormal condition for left HGS while removing each SNP for right HGS, almost SNPs caused bias (online supplemental table S3, online supplemental figures S3 and S4). The MR-heterogeneity test showed the presence of heterogeneity (online supplemental table S4). Egger regression showed no obvious pleiotropy. Using MR-PRESSO for further discovery of pleiotropy, the result remained stable (β=−0.195, p=0.023) (online supplemental table S5). Funnel plots are also shown in the supplementary materials (online supplemental figures S5 and S6).

10.1136/gpsych-2022-100807.supp3 Supplementary data

10.1136/gpsych-2022-100807.supp4 Supplementary data

10.1136/gpsych-2022-100807.supp5 Supplementary data

10.1136/gpsych-2022-100807.supp6 Supplementary data

**Table 2:** Correlation between genetically predicted HGS and potential confounders

Confounder	Outcome	SNP	OR	95% CI	P value
Alcoholic drinks per day	Left HGS	34	1.047	(0.959 to 1.144)	0.305
	Right HGS	34	1.054	(0.966 to 1.151)	0.239
Waist circumference	Left HGS	17	1.015	(0.960 to 1.073)	0.610
	Right HGS	17	1.027	(0.976 to 1.081)	0.306
Triglycerides	Left HGS	12	0.998	(0.981 to 1.014)	0.767
	Right HGS	12	1.000	(0.986 to 1.014)	0.978
HDL-C	Left HGS	4	0.997	(0.981 to 1.014)	0.722
	Right HGS	4	0.998	(0.984 to 1.013)	0.804
Glucose	Left HGS	22	0.995	(0.953 to 1.038)	0.808
	Right HGS	22	0.995	(0.960 to 1.032)	0.804
DBP	Left HGS	451	1.001	(0.999 to 1.002)	0.592
	Right HGS	451	1.000	(0.998 to 1.002)	0.785
SBP	Left HGS	446	1.000	(0.999 to 1.001)	0.755
	Right HGS	446	1.000	(0.999 to 1.001)	0.642
Exercise	Left HGS	13	1.142	(0.898 to 1.454)	0.280
	Right HGS	13	1.045	(0.841 to 1.298)	0.691
Frailty	Left HGS	15	0.882	(0.796 to 0.977)	0.016
	Right HGS	15	0.871	(0.779 to 0.974)	0.016

CI, confidence interval; DBP, diastolic blood pressure; HDL-C, high-density lipoprotein cholesterol; HGS, handgrip strength; OR, odds ratio; SBP, systolic blood pressure; SNP, single-nucleotide polymorphism.

## Discussion

### Main findings

Using data from 461 089 participants for HGS and 173 005 subjects (59 851 cases and 113 154 controls) for MDD, our MR study demonstrated that genetically predicted increased left HGS was causally associated with a 21.95% lower risk of MDD. Furthermore, we observed that genetic predisposition towards HGS was not correlated with established potential risk factors for MDD, including alcohol drinks per day, waist circumference, triglycerides, HDL-C, glucose, exercise and BP.

Previous outcomes reported by observational studies on the relationship between HGS and MDD were inconsistent. A cohort study of 7364 subjects from Korea concluded that HGS was not associated with MDD in adults after adjustment for age, sex, exercise and so on (adjusted OR=1.116, 95% CI: 0.585 to 2.128, p=0.897).^6^ However, another meta-analysis and systematic review involving 33 030 participants found a strong correlation after controlling for covariances (adjusted OR=1.821, 95% CI: 1.160 to 2.590, p=0.002).^22^ Discrepant findings in previous work may result from the relatively limited sample sizes of patients with MDD, leading to insufficient statistical power to precisely evaluate causality between HGS and MDD risk. On the other hand, few studies managed to control confounders like waist circumference and alcoholic drinks per day.^18^ More importantly, no prospective large-scale longitudinal cohort study has been conducted to date. To address these problems, in this study we examined the causal relationship between HGS and MDD at the gene level after removing the interference of several confounding factors.

### Implications

#### Special findings

Unexpectedly, we found that left HGS but not right HGS was associated with MDD. Among various factors that determine HGS, handedness may be the most important one. A previous study reported that left-handedness with a stronger left handgrip is associated with a 5.2% higher risk of being depressed.^23^ Handedness has been associated with brain lateralisation, which could be observed in patients with MDD.^24^ Moreover, a clinical trial using repetitive transcranial magnetic stimulation on 310 patients with MDD showed that targeting the left dorsolateral prefrontal cortex is more effective for left-handed than right-handed individuals.^25^ Left-handedness seems to be more clinically related to MDD.

#### Possible mechanisms

Potential mechanisms for increasing HGS on the reduced risk of MDD can be generally divided into two aspects. First, immunological reactions, including increased oxidative stress and inflammation, play an important role in their relationship. Oxidative stress, including reactive oxygen and nitrogen species (ROS/RNS), accumulates in the body as one ages and impairs muscle, causing the decline of HGS.^26^ ROS/RNS have also been related to MDD.^27^ Inflammatory factors, such as C reactive protein and interleukin-6, can both accelerate the catabolism of muscle^28^ and induce depressive symptoms.^29^ These biochemical processes affect movement and quality of life, indirectly causing low mood and even depressive symptoms. Second, HGS and MDD were both associated with hippocampal volume and white matter hyperintensities.^30^ Given that hippocampal volume reduction aggravates MDD in older depressed subjects, it is possible that decreased HGS correlates with cognitive dysfunction, a relevant symptom of MDD.^30^

#### Strength of the work

To the very best of our knowledge, this is the first MR study to assess the causality between genetically predicted HGS and MDD, with results highlighting the importance of HGS in the screening and clinical assessment of MDD. With large sample sizes (n=6 4 094) and robustly associated IVs, our two-sample MR provides adequate statistical power as well as a relatively precise estimation of a causal effect. Second, we performed additional MR analyses to investigate whether HGS-associated SNPs are linked to several confounding factors associated with the risk of MDD, namely, alcoholic drinks per day, waist circumference, triglycerides, HDL-C, glucose and BP. It also indicates that the causality between HGS and MDD was more likely due to the characteristics of HGS itself.

### Limitations of this study

Several limitations in our study cannot be ignored. First, although we have used the most comprehensive set of genetic variants known to date, it merely explained a limited part of the HGS variation across individuals. Unknown HGS-related SNPs could also play an important role in the development of MDD. Second, all three MR assumptions cannot be fully examined in our study, and potential violations against the assumptions may occur. It is possible that some of the genetic variants were also associated with confounders of HGS and MDD in our study, and caution is needed when considering the gross effect. For instance, low grip strength is associated with impaired quality of life and is a recognised marker of frailty, predicting physical decline and functional limitations in daily life.^21^ Also, despite the genetic factors being estimated to account for about 23.9% of the variation, HGS is also influenced by environmental factors and its interaction with genes. Third, all participants in our study were European, and it remains unclear whether our finding applies to other populations.

In conclusion, our results indicated that people with higher HGS have a lower risk of MDD and that HGS may be useful in the screening and assessment of MDD. The association between HGS and MDD and its mechanism has not yet been fully investigated, thus warranting further exploration.

## Data Availability

No data are available.
