# Supplementary material for: Handgrip strength and the risk of major depressive disorder: a two-sample Mendelian randomisation study
Source: Gen Psychiatr. 2022 Sep 27;35(5):e100807. doi: 10.1136/gpsych-2022-100807 (PMC9516288; doi:10.1136/gpsych-2022-100807)
Supplement: Supplementary data [file gpsych-2022-100807supp001.pdf]

Table S1. Detailed information of selected SNPs.

	exposure	outcome	SNP	b	se	p
1	Hand grip strength (right) || id:ukb-b-10215	Major Depressive Disorder || id:ieu-a-1188	rs10278546	-0.027892671	0.939194153	0.976307496
2	Hand grip strength (right) || id:ukb-b-10215	Major Depressive Disorder || id:ieu-a-1188	rs1043515	1.1715637	0.578532119	0.042860947
3	Hand grip strength (right) || id:ukb-b-10215	Major Depressive Disorder || id:ieu-a-1188	rs1047891	-0.360917547	0.898138685	0.687793999
4	Hand grip strength (right) || id:ukb-b-10215	Major Depressive Disorder || id:ieu-a-1188	rs10483727	0.022175575	0.898200605	0.980303118
5	Hand grip strength (right) || id:ukb-b-10215	Major Depressive Disorder || id:ieu-a-1188	rs10520770	0.894651678	0.672798501	0.183601698
6	Hand grip strength (right) || id:ukb-b-10215	Major Depressive Disorder || id:ieu-a-1188	rs10761411	0.512925419	0.965427267	0.595215275
7	Hand grip strength (right) || id:ukb-b-10215	Major Depressive Disorder || id:ieu-a-1188	rs10770125	1.30314927	1.006951519	0.195612598
8	Hand grip strength (right) || id:ukb-b-10215	Major Depressive Disorder || id:ieu-a-1188	rs10784502	-0.87218352	0.710131509	0.219371899
9	Hand grip strength (right) || id:ukb-b-10215	Major Depressive Disorder || id:ieu-a-1188	rs10798483	-0.495980232	0.544515898	0.362366742
10	Hand grip strength (right) || id:ukb-b-10215	Major Depressive Disorder || id:ieu-a-1188	rs10799428	0.013930133	0.724294509	0.984655478
11	Hand grip strength (right) || id:ukb-b-10215	Major Depressive Disorder || id:ieu-a-1188	rs11022513	-0.292747222	0.879427265	0.739221885
12	Hand grip strength (right) || id:ukb-b-10215	Major Depressive Disorder || id:ieu-a-1188	rs11039348	2.160105645	0.870139376	0.013047053
13	Hand grip strength (right) || id:ukb-b-10215	Major Depressive Disorder || id:ieu-a-1188	rs11243202	0.696243276	0.678822458	0.305050323
14	Hand grip strength (right) || id:ukb-b-10215	Major Depressive Disorder || id:ieu-a-1188	rs1125	-1.794255463	0.827484447	0.030133945
15	Hand grip strength (right) || id:ukb-b-10215	Major Depressive Disorder || id:ieu-a-1188	rs113315602	-0.586996445	2.174617563	0.787213426
16	Hand grip strength (right) || id:ukb-b-10215	Major Depressive Disorder || id:ieu-a-1188	rs113851275	2.853193837	0.951112802	0.002701144
17	Hand grip strength (right) || id:ukb-b-10215	Major Depressive Disorder || id:ieu-a-1188	rs114924396	0.669162514	0.915027895	0.464594186
18	Hand grip strength (right) || id:ukb-b-10215	Major Depressive Disorder || id:ieu-a-1188	rs11642954	0.272628451	0.750466199	0.716396637
19	Hand grip strength (right) || id:ukb-b-10215	Major Depressive Disorder || id:ieu-a-1188	rs116922558	-1.608147122	0.978596749	0.100316845
20	Hand grip strength (right) || id:ukb-b-10215	Major Depressive Disorder || id:ieu-a-1188	rs11998884	0.051869886	1.130119412	0.963391784
21	Hand grip strength (right) || id:ukb-b-10215	Major Depressive Disorder || id:ieu-a-1188	rs12052508	-0.62130665	0.928720687	0.503500622
22	Hand grip strength (right) || id:ukb-b-10215	Major Depressive Disorder || id:ieu-a-1188	rs12316046	-0.265309233	0.506029152	0.600072441
23	Hand grip strength (right) || id:ukb-b-10215	Major Depressive Disorder || id:ieu-a-1188	rs12412806	1.461990233	0.967477369	0.130753712
24	Hand grip strength (right) || id:ukb-b-10215	Major Depressive Disorder || id:ieu-a-1188	rs12522139	0.611593709	0.91798463	0.505260753
25	Hand grip strength (right) || id:ukb-b-10215	Major Depressive Disorder || id:ieu-a-1188	rs12616285	2.266876713	0.99840419	0.023177263
26	Hand grip strength (right) || id:ukb-b-10215	Major Depressive Disorder || id:ieu-a-1188	rs12763284	-1.572872264	0.823138743	0.056027359
27	Hand grip strength (right) || id:ukb-b-10215	Major Depressive Disorder || id:ieu-a-1188	rs12790261	-0.447458231	0.712893513	0.530223488
28	Hand grip strength (right) || id:ukb-b-10215	Major Depressive Disorder || id:ieu-a-1188	rs12823922	-1.120951288	0.84060839	0.182367776
29	Hand grip strength (right) || id:ukb-b-10215	Major Depressive Disorder || id:ieu-a-1188	rs12889267	-1.253145499	0.887087586	0.157758255
30	Hand grip strength (right) || id:ukb-b-10215	Major Depressive Disorder || id:ieu-a-1188	rs12899474	-0.959943333	0.866126401	0.267724578
31	Hand grip strength (right) || id:ukb-b-10215	Major Depressive Disorder || id:ieu-a-1188	rs12914702	-0.324349649	1.113296471	0.770790023
32	Hand grip strength (right) || id:ukb-b-10215	Major Depressive Disorder || id:ieu-a-1188	rs13106087	0.696305867	0.851004572	0.413233653
33	Hand grip strength (right) || id:ukb-b-10215	Major Depressive Disorder || id:ieu-a-1188	rs13107325	0.785512727	0.6	0.190470717
34	Hand grip strength (right) || id:ukb-b-10215	Major Depressive Disorder || id:ieu-a-1188	rs13146142	-0.802940837	0.591437144	0.174587546
35	Hand grip strength (right) || id:ukb-b-10215	Major Depressive Disorder || id:ieu-a-1188	rs13169333	-1.786858655	0.957933791	0.062135745
36	Hand grip strength (right) || id:ukb-b-10215	Major Depressive Disorder || id:ieu-a-1188	rs13355365	0.354889653	0.956763729	0.710691785

37	Hand grip strength (right) || id:ukb-b-10215	Major Depressive Disorder || id:ieu-a-1188	rs13356200	-0.901687653	0.901210989	0.317054611
38	Hand grip strength (right) || id:ukb-b-10215	Major Depressive Disorder || id:ieu-a-1188	rs143384	-0.321258295	0.351527408	0.360773286
39	Hand grip strength (right) || id:ukb-b-10215	Major Depressive Disorder || id:ieu-a-1188	rs1442883	-0.339347767	0.866379757	0.695291135
40	Hand grip strength (right) || id:ukb-b-10215	Major Depressive Disorder || id:ieu-a-1188	rs1486925	0.272503358	0.892032575	0.759996121
41	Hand grip strength (right) || id:ukb-b-10215	Major Depressive Disorder || id:ieu-a-1188	rs150330307	0.445147945	0.730440013	0.542243189
42	Hand grip strength (right) || id:ukb-b-10215	Major Depressive Disorder || id:ieu-a-1188	rs1550115	-1.426090712	0.60195242	0.017831012
43	Hand grip strength (right) || id:ukb-b-10215	Major Depressive Disorder || id:ieu-a-1188	rs1556659	0.319613904	0.479055576	0.504660358
44	Hand grip strength (right) || id:ukb-b-10215	Major Depressive Disorder || id:ieu-a-1188	rs1641457	0.122491318	0.719061243	0.864735728
45	Hand grip strength (right) || id:ukb-b-10215	Major Depressive Disorder || id:ieu-a-1188	rs1840753	1.37346136	0.972069944	0.157677948
46	Hand grip strength (right) || id:ukb-b-10215	Major Depressive Disorder || id:ieu-a-1188	rs1885690	-1.845233843	0.95242177	0.05269494
47	Hand grip strength (right) || id:ukb-b-10215	Major Depressive Disorder || id:ieu-a-1188	rs1892425	-1.014123521	0.8292386	0.221345769
48	Hand grip strength (right) || id:ukb-b-10215	Major Depressive Disorder || id:ieu-a-1188	rs1952256	0.614046403	0.849721179	0.469898273
49	Hand grip strength (right) || id:ukb-b-10215	Major Depressive Disorder || id:ieu-a-1188	rs2147461	-1.533622737	0.893528619	0.086094953
50	Hand grip strength (right) || id:ukb-b-10215	Major Depressive Disorder || id:ieu-a-1188	rs2165241	0.301815718	0.653135869	0.644007817
51	Hand grip strength (right) || id:ukb-b-10215	Major Depressive Disorder || id:ieu-a-1188	rs2194411	-0.560237623	0.861157592	0.515328354
52	Hand grip strength (right) || id:ukb-b-10215	Major Depressive Disorder || id:ieu-a-1188	rs2194747	-0.507294498	1.00494759	0.613701444
53	Hand grip strength (right) || id:ukb-b-10215	Major Depressive Disorder || id:ieu-a-1188	rs2208562	-0.640268648	0.700595073	0.360773286
54	Hand grip strength (right) || id:ukb-b-10215	Major Depressive Disorder || id:ieu-a-1188	rs2226685	0.977499927	0.900002903	0.277431423
55	Hand grip strength (right) || id:ukb-b-10215	Major Depressive Disorder || id:ieu-a-1188	rs2244621	-1.000112111	0.991746941	0.313245782
56	Hand grip strength (right) || id:ukb-b-10215	Major Depressive Disorder || id:ieu-a-1188	rs2273555	-0.453469125	0.725189456	0.531766798
57	Hand grip strength (right) || id:ukb-b-10215	Major Depressive Disorder || id:ieu-a-1188	rs2296316	-0.85281731	0.974026369	0.381269959
58	Hand grip strength (right) || id:ukb-b-10215	Major Depressive Disorder || id:ieu-a-1188	rs2322754	0.427482866	0.888721779	0.630510348
59	Hand grip strength (right) || id:ukb-b-10215	Major Depressive Disorder || id:ieu-a-1188	rs2341184	1.083354869	0.874699322	0.215513879
60	Hand grip strength (right) || id:ukb-b-10215	Major Depressive Disorder || id:ieu-a-1188	rs2362972	2.485251366	0.933642525	0.007770384
61	Hand grip strength (right) || id:ukb-b-10215	Major Depressive Disorder || id:ieu-a-1188	rs2389763	0.480651922	0.972838587	0.621255611
62	Hand grip strength (right) || id:ukb-b-10215	Major Depressive Disorder || id:ieu-a-1188	rs2431112	-4.252022914	0.716075904	2.89E-09
63	Hand grip strength (right) || id:ukb-b-10215	Major Depressive Disorder || id:ieu-a-1188	rs246181	0.010304174	0.86559388	0.990502074
64	Hand grip strength (right) || id:ukb-b-10215	Major Depressive Disorder || id:ieu-a-1188	rs248831	-0.970272672	0.950465576	0.307330552
65	Hand grip strength (right) || id:ukb-b-10215	Major Depressive Disorder || id:ieu-a-1188	rs2587505	0.571195142	0.938339247	0.542703495
66	Hand grip strength (right) || id:ukb-b-10215	Major Depressive Disorder || id:ieu-a-1188	rs2631360	0.456427824	0.706600002	0.518312147
67	Hand grip strength (right) || id:ukb-b-10215	Major Depressive Disorder || id:ieu-a-1188	rs2717351	1.801362622	0.759622538	0.017721237
68	Hand grip strength (right) || id:ukb-b-10215	Major Depressive Disorder || id:ieu-a-1188	rs2854152	-0.236250569	0.891638613	0.791038547
69	Hand grip strength (right) || id:ukb-b-10215	Major Depressive Disorder || id:ieu-a-1188	rs2894602	-0.394937433	0.937512336	0.673564467
70	Hand grip strength (right) || id:ukb-b-10215	Major Depressive Disorder || id:ieu-a-1188	rs3118914	0.24181258	0.493806839	0.624352837
71	Hand grip strength (right) || id:ukb-b-10215	Major Depressive Disorder || id:ieu-a-1188	rs34030812	-1.928448893	0.898277717	0.031806876
72	Hand grip strength (right) || id:ukb-b-10215	Major Depressive Disorder || id:ieu-a-1188	rs34845616	-0.030632278	1.061759703	0.97698384
73	Hand grip strength (right) || id:ukb-b-10215	Major Depressive Disorder || id:ieu-a-1188	rs35175534	3.326041851	3.341639645	0.319574677
74	Hand grip strength (right) || id:ukb-b-10215	Major Depressive Disorder || id:ieu-a-1188	rs35304341	-0.328914617	1.021965253	0.747569966

75	Hand grip strength (right) || id:ukb-b-10215	Major Depressive Disorder || id:ieu-a-1188	rs35457492	1.247636076	0.947368295	0.187855597
76	Hand grip strength (right) || id:ukb-b-10215	Major Depressive Disorder || id:ieu-a-1188	rs35701422	-0.74800747	0.943481978	0.427885118
77	Hand grip strength (right) || id:ukb-b-10215	Major Depressive Disorder || id:ieu-a-1188	rs35833641	0.35730224	0.921603025	0.698240668
78	Hand grip strength (right) || id:ukb-b-10215	Major Depressive Disorder || id:ieu-a-1188	rs36065733	0.556031897	0.884781855	0.529716045
79	Hand grip strength (right) || id:ukb-b-10215	Major Depressive Disorder || id:ieu-a-1188	rs3771498	-0.769690336	0.557889905	0.167695799
80	Hand grip strength (right) || id:ukb-b-10215	Major Depressive Disorder || id:ieu-a-1188	rs3848369	-0.513536721	0.933133147	0.582088967
81	Hand grip strength (right) || id:ukb-b-10215	Major Depressive Disorder || id:ieu-a-1188	rs4121165	-1.060087485	0.81808468	0.195038772
82	Hand grip strength (right) || id:ukb-b-10215	Major Depressive Disorder || id:ieu-a-1188	rs4369779	0.738260768	0.569668081	0.194993063
83	Hand grip strength (right) || id:ukb-b-10215	Major Depressive Disorder || id:ieu-a-1188	rs4549685	-0.278298168	0.864647646	0.747556412
84	Hand grip strength (right) || id:ukb-b-10215	Major Depressive Disorder || id:ieu-a-1188	rs4553566	0.752054708	0.849297072	0.375885655
85	Hand grip strength (right) || id:ukb-b-10215	Major Depressive Disorder || id:ieu-a-1188	rs4737446	-1.656183037	0.847391592	0.050648226
86	Hand grip strength (right) || id:ukb-b-10215	Major Depressive Disorder || id:ieu-a-1188	rs4751671	0.389260555	0.971675655	0.688709123
87	Hand grip strength (right) || id:ukb-b-10215	Major Depressive Disorder || id:ieu-a-1188	rs4752689	-1.18859604	0.913961911	0.193433938
88	Hand grip strength (right) || id:ukb-b-10215	Major Depressive Disorder || id:ieu-a-1188	rs4768725	-1.015303115	0.949338444	0.284851237
89	Hand grip strength (right) || id:ukb-b-10215	Major Depressive Disorder || id:ieu-a-1188	rs4784329	0.824801098	0.599893519	0.169158573
90	Hand grip strength (right) || id:ukb-b-10215	Major Depressive Disorder || id:ieu-a-1188	rs4785574	-1.465273829	0.770943153	0.057351149
91	Hand grip strength (right) || id:ukb-b-10215	Major Depressive Disorder || id:ieu-a-1188	rs4793658	-1.389782528	1.141126643	0.223260514
92	Hand grip strength (right) || id:ukb-b-10215	Major Depressive Disorder || id:ieu-a-1188	rs4927015	0.114777548	0.635576997	0.856691013
93	Hand grip strength (right) || id:ukb-b-10215	Major Depressive Disorder || id:ieu-a-1188	rs56074046	-2.877591859	0.929715706	0.00196726
94	Hand grip strength (right) || id:ukb-b-10215	Major Depressive Disorder || id:ieu-a-1188	rs56144131	1.025233359	0.856924254	0.231536322
95	Hand grip strength (right) || id:ukb-b-10215	Major Depressive Disorder || id:ieu-a-1188	rs56365901	-0.210668359	0.792326354	0.79032713
96	Hand grip strength (right) || id:ukb-b-10215	Major Depressive Disorder || id:ieu-a-1188	rs58670122	-0.952780677	0.915044542	0.297764329
97	Hand grip strength (right) || id:ukb-b-10215	Major Depressive Disorder || id:ieu-a-1188	rs600038	0.462454101	0.934840643	0.620820635
98	Hand grip strength (right) || id:ukb-b-10215	Major Depressive Disorder || id:ieu-a-1188	rs6006984	1.038674784	0.970996339	0.284754366
99	Hand grip strength (right) || id:ukb-b-10215	Major Depressive Disorder || id:ieu-a-1188	rs61389091	0.086065481	0.8977434	0.923624864
100	Hand grip strength (right) || id:ukb-b-10215	Major Depressive Disorder || id:ieu-a-1188	rs62037412	0.761517569	0.900354354	0.397665654
101	Hand grip strength (right) || id:ukb-b-10215	Major Depressive Disorder || id:ieu-a-1188	rs62234790	-0.963843697	0.859739622	0.262250444
102	Hand grip strength (right) || id:ukb-b-10215	Major Depressive Disorder || id:ieu-a-1188	rs62509875	0	0.811848435	1
103	Hand grip strength (right) || id:ukb-b-10215	Major Depressive Disorder || id:ieu-a-1188	rs635538	-2.350618115	0.64562182	0.00027173
104	Hand grip strength (right) || id:ukb-b-10215	Major Depressive Disorder || id:ieu-a-1188	rs645144	-0.103638611	0.989879062	0.916615246
105	Hand grip strength (right) || id:ukb-b-10215	Major Depressive Disorder || id:ieu-a-1188	rs6473015	0.260753301	0.906286818	0.773564071
106	Hand grip strength (right) || id:ukb-b-10215	Major Depressive Disorder || id:ieu-a-1188	rs6693965	0.867571226	0.719955695	0.228190104
107	Hand grip strength (right) || id:ukb-b-10215	Major Depressive Disorder || id:ieu-a-1188	rs6711390	-0.723328283	0.7010244	0.302158262
108	Hand grip strength (right) || id:ukb-b-10215	Major Depressive Disorder || id:ieu-a-1188	rs6715064	-1.140406111	0.944494142	0.227268402
109	Hand grip strength (right) || id:ukb-b-10215	Major Depressive Disorder || id:ieu-a-1188	rs6792762	-0.921875257	0.89943544	0.305387356
110	Hand grip strength (right) || id:ukb-b-10215	Major Depressive Disorder || id:ieu-a-1188	rs6882168	-1.110597903	0.934772663	0.234796332
111	Hand grip strength (right) || id:ukb-b-10215	Major Depressive Disorder || id:ieu-a-1188	rs6962338	-1.184246737	0.977944645	0.225912738
112	Hand grip strength (right) || id:ukb-b-10215	Major Depressive Disorder || id:ieu-a-1188	rs6977081	0.552465008	0.65318818	0.397665654

113	Hand grip strength (right) || id:ukb-b-10215	Major Depressive Disorder || id:ieu-a-1188	rs7034200	1.622801465	0.909222741	0.074290066
114	Hand grip strength (right) || id:ukb-b-10215	Major Depressive Disorder || id:ieu-a-1188	rs71298370	0.825078015	0.945817319	0.383020769
115	Hand grip strength (right) || id:ukb-b-10215	Major Depressive Disorder || id:ieu-a-1188	rs7148603	2.138450677	0.913376452	0.019218839
116	Hand grip strength (right) || id:ukb-b-10215	Major Depressive Disorder || id:ieu-a-1188	rs7196917	-1.161907012	0.756000756	0.124314738
117	Hand grip strength (right) || id:ukb-b-10215	Major Depressive Disorder || id:ieu-a-1188	rs7206195	-1.468882783	0.920299456	0.11046821
118	Hand grip strength (right) || id:ukb-b-10215	Major Depressive Disorder || id:ieu-a-1188	rs721101	-0.581476439	0.940110722	0.536233211
119	Hand grip strength (right) || id:ukb-b-10215	Major Depressive Disorder || id:ieu-a-1188	rs7214252	-2.53793366	0.946877257	0.00735519
120	Hand grip strength (right) || id:ukb-b-10215	Major Depressive Disorder || id:ieu-a-1188	rs7249	2.664816444	1.025718416	0.009376849
121	Hand grip strength (right) || id:ukb-b-10215	Major Depressive Disorder || id:ieu-a-1188	rs7266065	1.368081289	0.845171605	0.105511314
122	Hand grip strength (right) || id:ukb-b-10215	Major Depressive Disorder || id:ieu-a-1188	rs7301953	-1.369567666	0.736587606	0.062978794
123	Hand grip strength (right) || id:ukb-b-10215	Major Depressive Disorder || id:ieu-a-1188	rs7451021	0.639298081	0.538087069	0.234796332
124	Hand grip strength (right) || id:ukb-b-10215	Major Depressive Disorder || id:ieu-a-1188	rs75457267	0.790143891	0.998424945	0.428716225
125	Hand grip strength (right) || id:ukb-b-10215	Major Depressive Disorder || id:ieu-a-1188	rs7549184	0.037871212	0.909090909	0.96677107
126	Hand grip strength (right) || id:ukb-b-10215	Major Depressive Disorder || id:ieu-a-1188	rs7565148	-0.666960999	0.763203911	0.382174783
127	Hand grip strength (right) || id:ukb-b-10215	Major Depressive Disorder || id:ieu-a-1188	rs7657558	-1.445088807	0.829798145	0.081596957
128	Hand grip strength (right) || id:ukb-b-10215	Major Depressive Disorder || id:ieu-a-1188	rs76749769	1.039185116	1.025534421	0.310911725
129	Hand grip strength (right) || id:ukb-b-10215	Major Depressive Disorder || id:ieu-a-1188	rs76895963	0.530929397	1.036770612	0.608581587
130	Hand grip strength (right) || id:ukb-b-10215	Major Depressive Disorder || id:ieu-a-1188	rs7790322	-0.999986051	0.929907091	0.282213024
131	Hand grip strength (right) || id:ukb-b-10215	Major Depressive Disorder || id:ieu-a-1188	rs7871404	-0.661839206	0.846285978	0.434184317
132	Hand grip strength (right) || id:ukb-b-10215	Major Depressive Disorder || id:ieu-a-1188	rs7963801	0.878247024	0.718505509	0.221584866
133	Hand grip strength (right) || id:ukb-b-10215	Major Depressive Disorder || id:ieu-a-1188	rs79723785	0.886902944	1.656368207	0.592338478
134	Hand grip strength (right) || id:ukb-b-10215	Major Depressive Disorder || id:ieu-a-1188	rs8055199	0.859075806	1.015328107	0.397493283
135	Hand grip strength (right) || id:ukb-b-10215	Major Depressive Disorder || id:ieu-a-1188	rs823130	-1.332270408	0.726556445	0.066701839
136	Hand grip strength (right) || id:ukb-b-10215	Major Depressive Disorder || id:ieu-a-1188	rs852520	-0.28045189	0.965960018	0.771560054
137	Hand grip strength (right) || id:ukb-b-10215	Major Depressive Disorder || id:ieu-a-1188	rs911642	-0.764979212	0.961765756	0.426386465
138	Hand grip strength (right) || id:ukb-b-10215	Major Depressive Disorder || id:ieu-a-1188	rs9322822	-2.201752723	0.767154938	0.004104413
139	Hand grip strength (right) || id:ukb-b-10215	Major Depressive Disorder || id:ieu-a-1188	rs935728	0.502280347	0.879270875	0.56783261
140	Hand grip strength (right) || id:ukb-b-10215	Major Depressive Disorder || id:ieu-a-1188	rs9388051	-0.960070392	0.969302291	0.321941647
141	Hand grip strength (right) || id:ukb-b-10215	Major Depressive Disorder || id:ieu-a-1188	rs9396861	-0.623273574	0.893068542	0.485239007
142	Hand grip strength (right) || id:ukb-b-10215	Major Depressive Disorder || id:ieu-a-1188	rs9652468	0.438525074	0.734190953	0.550313635
143	Hand grip strength (right) || id:ukb-b-10215	Major Depressive Disorder || id:ieu-a-1188	rs9757079	-0.714579234	0.867149618	0.40990814
144	Hand grip strength (right) || id:ukb-b-10215	Major Depressive Disorder || id:ieu-a-1188	rs9853018	-0.735267756	0.774684488	0.342560098
145	Hand grip strength (right) || id:ukb-b-10215	Major Depressive Disorder || id:ieu-a-1188	All - Inverse v	-0.161072931	0.090504806	0.075122307
146	Hand grip strength (right) || id:ukb-b-10215	Major Depressive Disorder || id:ieu-a-1188	All - MR Egge	-0.016376757	0.341456848	0.961814376
147	Hand grip strength (left) || id:ukb-b-7478	Major Depressive Disorder || id:ieu-a-1188	rs10097417	-0.068056794	0.801197261	0.932306016
148	Hand grip strength (left) || id:ukb-b-7478	Major Depressive Disorder || id:ieu-a-1188	rs10176878	-0.077271815	0.795501939	0.922618454
149	Hand grip strength (left) || id:ukb-b-7478	Major Depressive Disorder || id:ieu-a-1188	rs10403906	-1.026364842	0.797424319	0.198059435
150	Hand grip strength (left) || id:ukb-b-7478	Major Depressive Disorder || id:ieu-a-1188	rs1044299	-0.571158458	0.5710207	0.317193771

151	Hand grip strength (left) || id:ukb-b-7478	Major Depressive Disorder || id:ieu-a-1188	rs10786706	-1.458549844	0.789431609	0.064660963
152	Hand grip strength (left) || id:ukb-b-7478	Major Depressive Disorder || id:ieu-a-1188	rs10821939	-0.737225552	0.865919339	0.394558846
153	Hand grip strength (left) || id:ukb-b-7478	Major Depressive Disorder || id:ieu-a-1188	rs10831903	-0.215578973	0.882990798	0.807117483
154	Hand grip strength (left) || id:ukb-b-7478	Major Depressive Disorder || id:ieu-a-1188	rs10934857	-0.947434786	1.001426764	0.344105261
155	Hand grip strength (left) || id:ukb-b-7478	Major Depressive Disorder || id:ieu-a-1188	rs10988217	0.988259471	0.890668294	0.267184049
156	Hand grip strength (left) || id:ukb-b-7478	Major Depressive Disorder || id:ieu-a-1188	rs11002322	-0.340786686	0.830894042	0.681699893
157	Hand grip strength (left) || id:ukb-b-7478	Major Depressive Disorder || id:ieu-a-1188	rs11076004	1.092184195	0.693505323	0.1152853
158	Hand grip strength (left) || id:ukb-b-7478	Major Depressive Disorder || id:ieu-a-1188	rs11111267	0.055388108	0.941315442	0.953078611
159	Hand grip strength (left) || id:ukb-b-7478	Major Depressive Disorder || id:ieu-a-1188	rs11121542	0.863071935	0.755071351	0.253024635
160	Hand grip strength (left) || id:ukb-b-7478	Major Depressive Disorder || id:ieu-a-1188	rs11125803	-1.357841387	0.644027693	0.034999974
161	Hand grip strength (left) || id:ukb-b-7478	Major Depressive Disorder || id:ieu-a-1188	rs11168357	0.404791331	0.976254175	0.678407487
162	Hand grip strength (left) || id:ukb-b-7478	Major Depressive Disorder || id:ieu-a-1188	rs11204664	-0.508523042	0.970891741	0.600439184
163	Hand grip strength (left) || id:ukb-b-7478	Major Depressive Disorder || id:ieu-a-1188	rs11243202	0.833412018	0.812559078	0.305050323
164	Hand grip strength (left) || id:ukb-b-7478	Major Depressive Disorder || id:ieu-a-1188	rs112485536	0.074130759	0.914828253	0.93541616
165	Hand grip strength (left) || id:ukb-b-7478	Major Depressive Disorder || id:ieu-a-1188	rs113315602	-0.687122703	2.54555051	0.787213426
166	Hand grip strength (left) || id:ukb-b-7478	Major Depressive Disorder || id:ieu-a-1188	rs113434679	-0.295013275	0.784413634	0.706847002
167	Hand grip strength (left) || id:ukb-b-7478	Major Depressive Disorder || id:ieu-a-1188	rs113918482	-2.128431557	0.950391542	0.02512137
168	Hand grip strength (left) || id:ukb-b-7478	Major Depressive Disorder || id:ieu-a-1188	rs116409670	0.780780485	0.964453017	0.418194357
169	Hand grip strength (left) || id:ukb-b-7478	Major Depressive Disorder || id:ieu-a-1188	rs11642954	0.307324076	0.845973083	0.716396637
170	Hand grip strength (left) || id:ukb-b-7478	Major Depressive Disorder || id:ieu-a-1188	rs116922558	-1.834860685	1.116557482	0.100316845
171	Hand grip strength (left) || id:ukb-b-7478	Major Depressive Disorder || id:ieu-a-1188	rs12316046	-0.246848104	0.470817903	0.600072441
172	Hand grip strength (left) || id:ukb-b-7478	Major Depressive Disorder || id:ieu-a-1188	rs12473732	-0.964412164	0.728139876	0.185341192
173	Hand grip strength (left) || id:ukb-b-7478	Major Depressive Disorder || id:ieu-a-1188	rs12528131	-1.699388755	0.90621984	0.060758338
174	Hand grip strength (left) || id:ukb-b-7478	Major Depressive Disorder || id:ieu-a-1188	rs12533765	-1.792967391	0.967391304	0.063824404
175	Hand grip strength (left) || id:ukb-b-7478	Major Depressive Disorder || id:ieu-a-1188	rs12673062	0.324641799	0.91001105	0.72128279
176	Hand grip strength (left) || id:ukb-b-7478	Major Depressive Disorder || id:ieu-a-1188	rs12790261	-0.468477303	0.746381242	0.530223488
177	Hand grip strength (left) || id:ukb-b-7478	Major Depressive Disorder || id:ieu-a-1188	rs12889267	-1.120711239	0.793338865	0.157758255
178	Hand grip strength (left) || id:ukb-b-7478	Major Depressive Disorder || id:ieu-a-1188	rs12906830	-0.248739403	0.747225579	0.739221885
179	Hand grip strength (left) || id:ukb-b-7478	Major Depressive Disorder || id:ieu-a-1188	rs12914702	-0.319237723	1.095750315	0.770790023
180	Hand grip strength (left) || id:ukb-b-7478	Major Depressive Disorder || id:ieu-a-1188	rs13091492	0.731302991	0.967091755	0.449536603
181	Hand grip strength (left) || id:ukb-b-7478	Major Depressive Disorder || id:ieu-a-1188	rs13106087	0.774546049	0.946627425	0.413233653
182	Hand grip strength (left) || id:ukb-b-7478	Major Depressive Disorder || id:ieu-a-1188	rs13107325	0.825897619	0.630847285	0.190470717
183	Hand grip strength (left) || id:ukb-b-7478	Major Depressive Disorder || id:ieu-a-1188	rs13146142	-0.826536522	0.608817459	0.174587546
184	Hand grip strength (left) || id:ukb-b-7478	Major Depressive Disorder || id:ieu-a-1188	rs13227429	-0.418793456	1.048014188	0.689446245
185	Hand grip strength (left) || id:ukb-b-7478	Major Depressive Disorder || id:ieu-a-1188	rs13337177	-0.518388084	0.86835342	0.550521918
186	Hand grip strength (left) || id:ukb-b-7478	Major Depressive Disorder || id:ieu-a-1188	rs13356200	-0.947162581	0.946661876	0.317054611
187	Hand grip strength (left) || id:ukb-b-7478	Major Depressive Disorder || id:ieu-a-1188	rs143002906	-0.472641937	0.945550764	0.617174454
188	Hand grip strength (left) || id:ukb-b-7478	Major Depressive Disorder || id:ieu-a-1188	rs143384	-0.353846044	0.387185591	0.360773286

189	Hand grip strength (left) || id:ukb-b-7478	Major Depressive Disorder || id:ieu-a-1188	rs1434095	0.320746823	0.876686552	0.714467604
190	Hand grip strength (left) || id:ukb-b-7478	Major Depressive Disorder || id:ieu-a-1188	rs1486925	0.247926175	0.811579812	0.759996121
191	Hand grip strength (left) || id:ukb-b-7478	Major Depressive Disorder || id:ieu-a-1188	rs150330307	0.471296787	0.773347457	0.542243189
192	Hand grip strength (left) || id:ukb-b-7478	Major Depressive Disorder || id:ieu-a-1188	rs1551042	-0.710028846	0.745872986	0.341125701
193	Hand grip strength (left) || id:ukb-b-7478	Major Depressive Disorder || id:ieu-a-1188	rs1556659	0.344039755	0.515666437	0.504660358
194	Hand grip strength (left) || id:ukb-b-7478	Major Depressive Disorder || id:ieu-a-1188	rs1641457	0.132934848	0.780367935	0.864735728
195	Hand grip strength (left) || id:ukb-b-7478	Major Depressive Disorder || id:ieu-a-1188	rs16870531	-0.142996964	0.830505447	0.863295514
196	Hand grip strength (left) || id:ukb-b-7478	Major Depressive Disorder || id:ieu-a-1188	rs17282763	-3.668670494	0.962016028	0.000137
197	Hand grip strength (left) || id:ukb-b-7478	Major Depressive Disorder || id:ieu-a-1188	rs17466480	0.490813211	0.693534064	0.479132335
198	Hand grip strength (left) || id:ukb-b-7478	Major Depressive Disorder || id:ieu-a-1188	rs17630248	-1.503146777	0.903745645	0.096264211
199	Hand grip strength (left) || id:ukb-b-7478	Major Depressive Disorder || id:ieu-a-1188	rs181766	-0.363754053	0.892813577	0.683696295
200	Hand grip strength (left) || id:ukb-b-7478	Major Depressive Disorder || id:ieu-a-1188	rs1884447	-0.838597088	0.957368725	0.381062921
201	Hand grip strength (left) || id:ukb-b-7478	Major Depressive Disorder || id:ieu-a-1188	rs1981612	0.812977443	0.888643248	0.360269101
202	Hand grip strength (left) || id:ukb-b-7478	Major Depressive Disorder || id:ieu-a-1188	rs2038760	-0.190659541	0.919643942	0.83576084
203	Hand grip strength (left) || id:ukb-b-7478	Major Depressive Disorder || id:ieu-a-1188	rs217181	-1.095354401	0.83644211	0.190351498
204	Hand grip strength (left) || id:ukb-b-7478	Major Depressive Disorder || id:ieu-a-1188	rs2359239	2.303539238	0.907584113	0.011145613
205	Hand grip strength (left) || id:ukb-b-7478	Major Depressive Disorder || id:ieu-a-1188	rs2431112	-4.956056155	0.834640937	2.89E-09
206	Hand grip strength (left) || id:ukb-b-7478	Major Depressive Disorder || id:ieu-a-1188	rs2532111	0.223879944	0.836152919	0.78889176
207	Hand grip strength (left) || id:ukb-b-7478	Major Depressive Disorder || id:ieu-a-1188	rs2587505	0.588161799	0.966211474	0.542703495
208	Hand grip strength (left) || id:ukb-b-7478	Major Depressive Disorder || id:ieu-a-1188	rs2631360	0.467406139	0.723595629	0.518312147
209	Hand grip strength (left) || id:ukb-b-7478	Major Depressive Disorder || id:ieu-a-1188	rs2800789	1.828455555	0.96849369	0.059034341
210	Hand grip strength (left) || id:ukb-b-7478	Major Depressive Disorder || id:ieu-a-1188	rs28542042	-0.072689621	0.808348698	0.928347931
211	Hand grip strength (left) || id:ukb-b-7478	Major Depressive Disorder || id:ieu-a-1188	rs2871960	-0.595967041	0.653546108	0.361822694
212	Hand grip strength (left) || id:ukb-b-7478	Major Depressive Disorder || id:ieu-a-1188	rs2974438	-0.566208368	0.992703628	0.568426773
213	Hand grip strength (left) || id:ukb-b-7478	Major Depressive Disorder || id:ieu-a-1188	rs3118903	0.206128028	0.550215788	0.707934729
214	Hand grip strength (left) || id:ukb-b-7478	Major Depressive Disorder || id:ieu-a-1188	rs34030812	-1.731330953	0.806459544	0.031806876
215	Hand grip strength (left) || id:ukb-b-7478	Major Depressive Disorder || id:ieu-a-1188	rs34722008	-1.93310031	0.978372152	0.048173657
216	Hand grip strength (left) || id:ukb-b-7478	Major Depressive Disorder || id:ieu-a-1188	rs34845616	-0.027680705	0.959453849	0.97698384
217	Hand grip strength (left) || id:ukb-b-7478	Major Depressive Disorder || id:ieu-a-1188	rs35175534	3.896636597	3.91491025	0.319574677
218	Hand grip strength (left) || id:ukb-b-7478	Major Depressive Disorder || id:ieu-a-1188	rs35236379	-1.222312143	0.930684255	0.18906577
219	Hand grip strength (left) || id:ukb-b-7478	Major Depressive Disorder || id:ieu-a-1188	rs3814877	-1.072600465	0.768610334	0.162863255
220	Hand grip strength (left) || id:ukb-b-7478	Major Depressive Disorder || id:ieu-a-1188	rs3819121	-0.759241787	0.659747593	0.24981187
221	Hand grip strength (left) || id:ukb-b-7478	Major Depressive Disorder || id:ieu-a-1188	rs4121165	-1.112298435	0.858376617	0.195038772
222	Hand grip strength (left) || id:ukb-b-7478	Major Depressive Disorder || id:ieu-a-1188	rs41271299	0.30670675	0.986356193	0.755839178
223	Hand grip strength (left) || id:ukb-b-7478	Major Depressive Disorder || id:ieu-a-1188	rs4308051	0.775125212	0.612771918	0.205889618
224	Hand grip strength (left) || id:ukb-b-7478	Major Depressive Disorder || id:ieu-a-1188	rs4335354	2.28861976	0.915469194	0.012421369
225	Hand grip strength (left) || id:ukb-b-7478	Major Depressive Disorder || id:ieu-a-1188	rs4498020	-0.691109768	0.844221877	0.412994562
226	Hand grip strength (left) || id:ukb-b-7478	Major Depressive Disorder || id:ieu-a-1188	rs4621706	-0.691660403	0.683363515	0.311470522

227	Hand grip strength (left) || id:ukb-b-7478	Major Depressive Disorder || id:ieu-a-1188	rs4677601	0	0.872076336	1
228	Hand grip strength (left) || id:ukb-b-7478	Major Depressive Disorder || id:ieu-a-1188	rs4737446	-1.632177619	0.83510914	0.050648226
229	Hand grip strength (left) || id:ukb-b-7478	Major Depressive Disorder || id:ieu-a-1188	rs4739739	1.839282764	0.972595083	0.058609871
230	Hand grip strength (left) || id:ukb-b-7478	Major Depressive Disorder || id:ieu-a-1188	rs4930236	-0.268423617	0.957725654	0.779268885
231	Hand grip strength (left) || id:ukb-b-7478	Major Depressive Disorder || id:ieu-a-1188	rs55681913	0.64611654	0.97286152	0.50660052
232	Hand grip strength (left) || id:ukb-b-7478	Major Depressive Disorder || id:ieu-a-1188	rs56060323	-0.264953882	0.94828014	0.779934302
233	Hand grip strength (left) || id:ukb-b-7478	Major Depressive Disorder || id:ieu-a-1188	rs56338231	0.350581728	0.829722504	0.672639019
234	Hand grip strength (left) || id:ukb-b-7478	Major Depressive Disorder || id:ieu-a-1188	rs58670122	-1.066374378	1.024139215	0.297764329
235	Hand grip strength (left) || id:ukb-b-7478	Major Depressive Disorder || id:ieu-a-1188	rs59116179	0.548054841	0.968059856	0.571300415
236	Hand grip strength (left) || id:ukb-b-7478	Major Depressive Disorder || id:ieu-a-1188	rs6006984	1.086081365	1.015313981	0.284754366
237	Hand grip strength (left) || id:ukb-b-7478	Major Depressive Disorder || id:ieu-a-1188	rs61389091	0.072553674	0.756802624	0.923624864
238	Hand grip strength (left) || id:ukb-b-7478	Major Depressive Disorder || id:ieu-a-1188	rs61818100	-1.540238949	0.892711015	0.084464416
239	Hand grip strength (left) || id:ukb-b-7478	Major Depressive Disorder || id:ieu-a-1188	rs62081464	-3.420340595	0.94103838	0.000278363
240	Hand grip strength (left) || id:ukb-b-7478	Major Depressive Disorder || id:ieu-a-1188	rs62253653	0.046747208	0.822545217	0.954678715
241	Hand grip strength (left) || id:ukb-b-7478	Major Depressive Disorder || id:ieu-a-1188	rs635538	-2.387239171	0.655680176	0.00027173
242	Hand grip strength (left) || id:ukb-b-7478	Major Depressive Disorder || id:ieu-a-1188	rs6433478	-0.121390257	0.894361547	0.892035999
243	Hand grip strength (left) || id:ukb-b-7478	Major Depressive Disorder || id:ieu-a-1188	rs6680160	1.688784935	0.814413126	0.038114435
244	Hand grip strength (left) || id:ukb-b-7478	Major Depressive Disorder || id:ieu-a-1188	rs6802071	-0.850494371	0.861348425	0.323447189
245	Hand grip strength (left) || id:ukb-b-7478	Major Depressive Disorder || id:ieu-a-1188	rs6882168	-1.079638695	0.908714789	0.234796332
246	Hand grip strength (left) || id:ukb-b-7478	Major Depressive Disorder || id:ieu-a-1188	rs6962338	-1.125575095	0.929493916	0.225912738
247	Hand grip strength (left) || id:ukb-b-7478	Major Depressive Disorder || id:ieu-a-1188	rs6977081	0.48159295	0.569395018	0.397665654
248	Hand grip strength (left) || id:ukb-b-7478	Major Depressive Disorder || id:ieu-a-1188	rs7026798	-0.242594081	0.971347672	0.802780689
249	Hand grip strength (left) || id:ukb-b-7478	Major Depressive Disorder || id:ieu-a-1188	rs7124681	0.901275771	0.686359464	0.189140831
250	Hand grip strength (left) || id:ukb-b-7478	Major Depressive Disorder || id:ieu-a-1188	rs71298370	0.920639402	1.055362857	0.383020769
251	Hand grip strength (left) || id:ukb-b-7478	Major Depressive Disorder || id:ieu-a-1188	rs7148603	2.079040457	0.88800112	0.019218839
252	Hand grip strength (left) || id:ukb-b-7478	Major Depressive Disorder || id:ieu-a-1188	rs7176095	1.211072897	1.024299065	0.237069604
253	Hand grip strength (left) || id:ukb-b-7478	Major Depressive Disorder || id:ieu-a-1188	rs7196917	-1.048174797	0.682000307	0.124314738
254	Hand grip strength (left) || id:ukb-b-7478	Major Depressive Disorder || id:ieu-a-1188	rs7197751	-0.021132364	0.898215299	0.981229855
255	Hand grip strength (left) || id:ukb-b-7478	Major Depressive Disorder || id:ieu-a-1188	rs723588	0.703682449	0.875656743	0.421624928
256	Hand grip strength (left) || id:ukb-b-7478	Major Depressive Disorder || id:ieu-a-1188	rs7516571	-0.79993666	0.949031668	0.39928563
257	Hand grip strength (left) || id:ukb-b-7478	Major Depressive Disorder || id:ieu-a-1188	rs75497896	0.657732178	0.85136436	0.439781345
258	Hand grip strength (left) || id:ukb-b-7478	Major Depressive Disorder || id:ieu-a-1188	rs755547	0.871654678	0.617597907	0.158137682
259	Hand grip strength (left) || id:ukb-b-7478	Major Depressive Disorder || id:ieu-a-1188	rs7571789	-0.802522792	0.61757463	0.193780968
260	Hand grip strength (left) || id:ukb-b-7478	Major Depressive Disorder || id:ieu-a-1188	rs76895963	0.531131651	1.037165563	0.608581587
261	Hand grip strength (left) || id:ukb-b-7478	Major Depressive Disorder || id:ieu-a-1188	rs772014	-0.471141856	0.753451751	0.531766798
262	Hand grip strength (left) || id:ukb-b-7478	Major Depressive Disorder || id:ieu-a-1188	rs7856625	-0.727524551	0.72778242	0.317482009
263	Hand grip strength (left) || id:ukb-b-7478	Major Depressive Disorder || id:ieu-a-1188	rs7963801	0.94882796	0.776248706	0.221584866
264	Hand grip strength (left) || id:ukb-b-7478	Major Depressive Disorder || id:ieu-a-1188	rs7970350	-1.125334254	0.779515516	0.148842262

265	Hand grip strength (left) || id:ukb-b-7478	Major Depressive Disorder || id:ieu-a-1188	rs8101782	1.622844949	0.994678991	0.102779312
266	Hand grip strength (left) || id:ukb-b-7478	Major Depressive Disorder || id:ieu-a-1188	rs8108461	0.189167707	0.913488444	0.835945227
267	Hand grip strength (left) || id:ukb-b-7478	Major Depressive Disorder || id:ieu-a-1188	rs821100	1.264647911	0.882551948	0.151873715
268	Hand grip strength (left) || id:ukb-b-7478	Major Depressive Disorder || id:ieu-a-1188	rs823130	-1.45636577	0.79423211	0.066701839
269	Hand grip strength (left) || id:ukb-b-7478	Major Depressive Disorder || id:ieu-a-1188	rs9371201	-1.841971972	0.888796559	0.03822501
270	Hand grip strength (left) || id:ukb-b-7478	Major Depressive Disorder || id:ieu-a-1188	rs9371881	0.042188057	0.875577301	0.961570293
271	Hand grip strength (left) || id:ukb-b-7478	Major Depressive Disorder || id:ieu-a-1188	rs9388769	0.490252164	0.596501942	0.41114601
272	Hand grip strength (left) || id:ukb-b-7478	Major Depressive Disorder || id:ieu-a-1188	rs9611273	-1.186563844	0.880542785	0.177807396
273	Hand grip strength (left) || id:ukb-b-7478	Major Depressive Disorder || id:ieu-a-1188	rs9866627	-0.032045529	0.897499183	0.971517299
274	Hand grip strength (left) || id:ukb-b-7478	Major Depressive Disorder || id:ieu-a-1188	rs9944324	0.186925329	0.935374945	0.841605717
275	Hand grip strength (left) || id:ukb-b-7478	Major Depressive Disorder || id:ieu-a-1188	rs999493	-1.398545601	0.637076286	0.028145232
276	Hand grip strength (left) || id:ukb-b-7478	Major Depressive Disorder || id:ieu-a-1188	All - Inverse v	-0.246735109	0.09339371	0.008244691
277	Hand grip strength (left) || id:ukb-b-7478	Major Depressive Disorder || id:ieu-a-1188	All - MR Egge	-0.00638563	0.360663959	0.985901813
