# Supplementary material for: Handgrip strength and the risk of major depressive disorder: a two-sample Mendelian randomisation study
Source: Gen Psychiatr. 2022 Sep 27;35(5):e100807. doi: 10.1136/gpsych-2022-100807 (PMC9516288; doi:10.1136/gpsych-2022-100807)
Supplement: Supplementary data [file gpsych-2022-100807supp002.pdf]

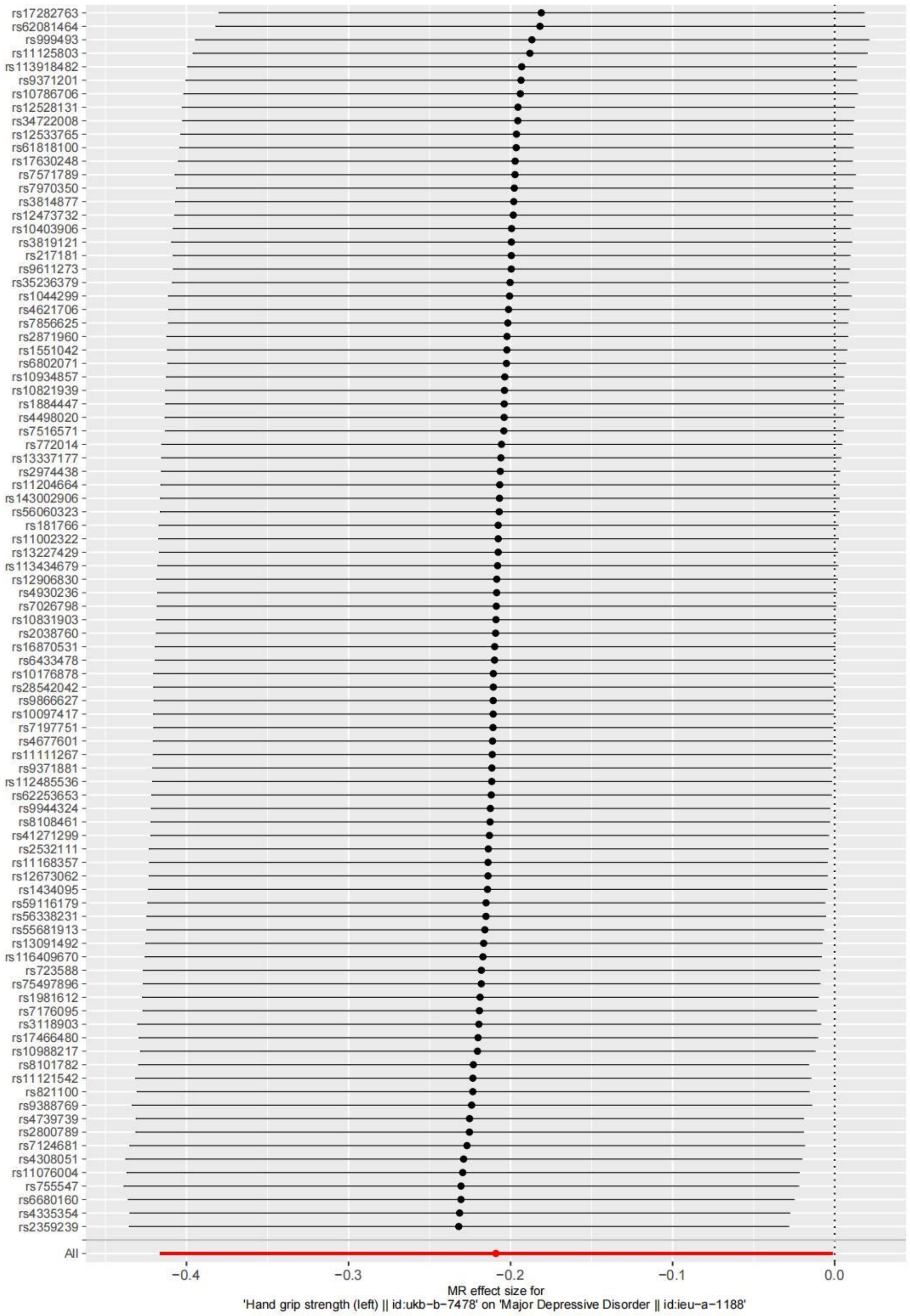

Figure S1. Forest plot of the effect of left handgrip strength (HGS) on major depressive disorder (MDD).

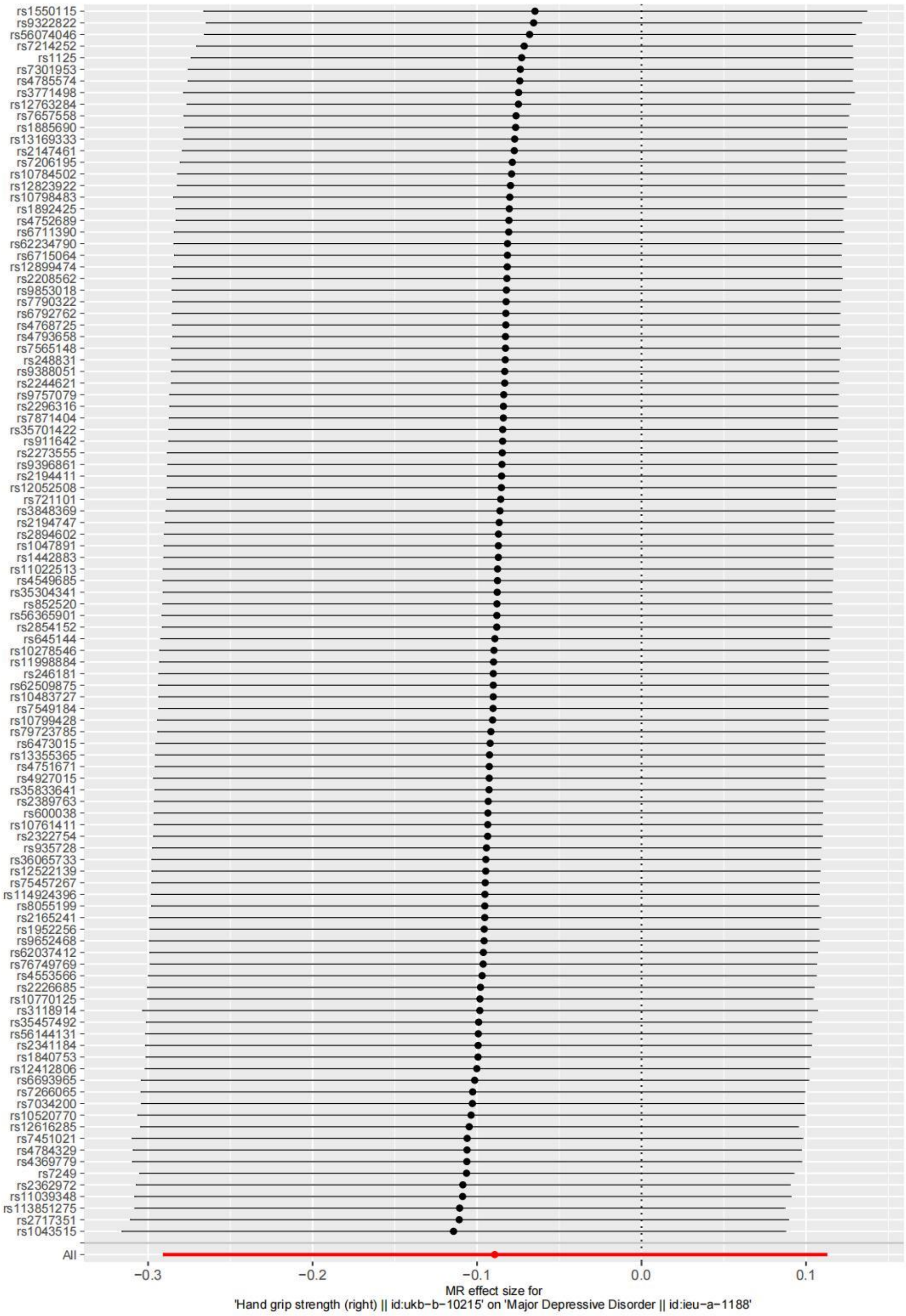

Figure S2. Forest plot of the effect of right HGS on MDD.

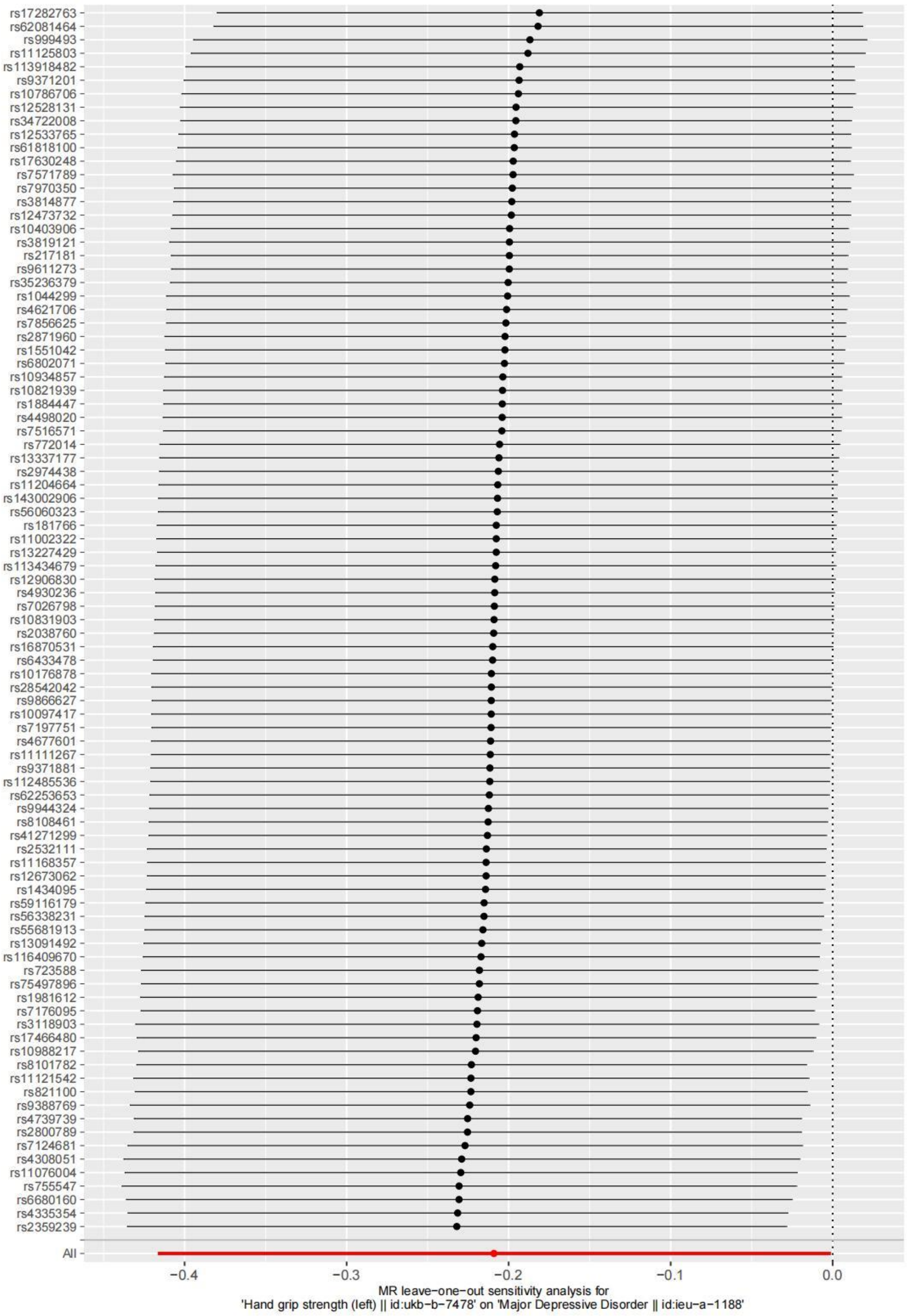

Figure S3. Leave-one-out plot of the effect of left HGS on MDD.

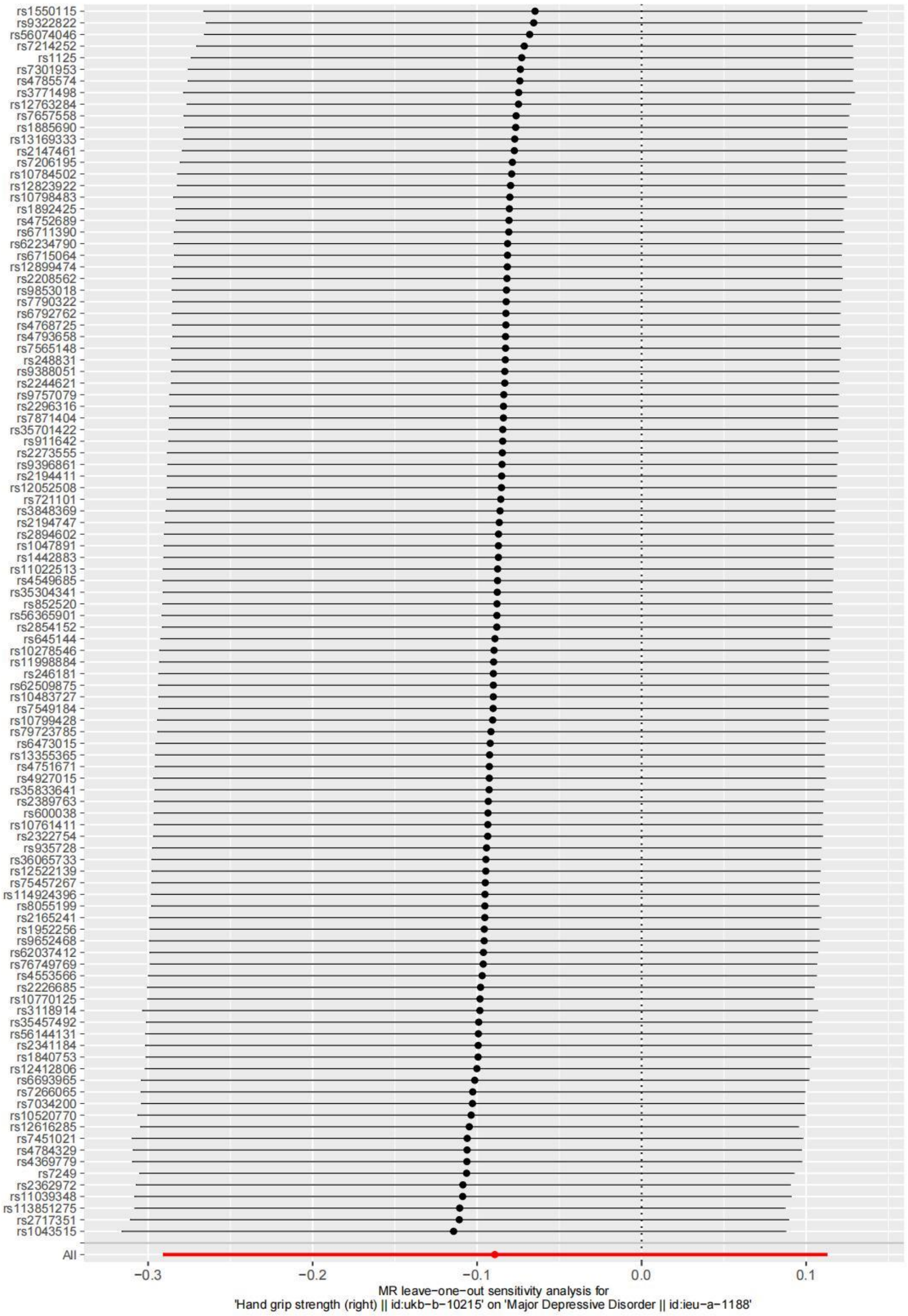

Figure S4. Leave-one-out plot of the effect of right HGS on MDD.

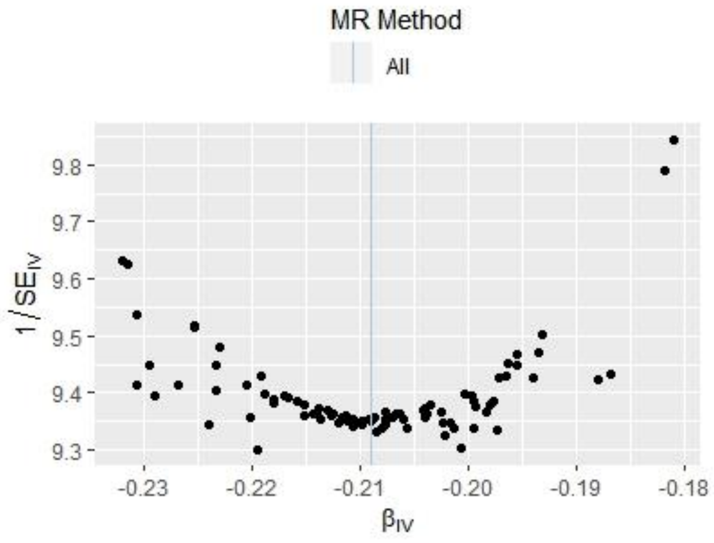

Figure S5. Funnel plot about the effect of left HGS on MDD.

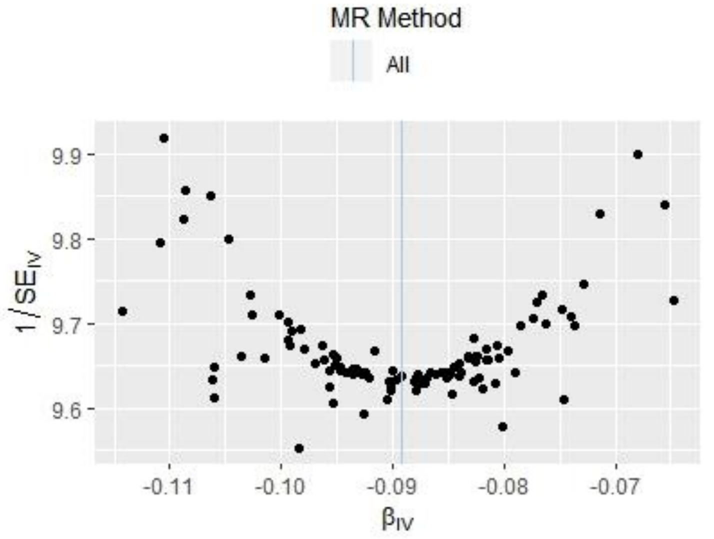

Figure S6. Funnel plot about the effect of right HGS on MDD.
