# Supplementary material for: Handgrip strength and the risk of major depressive disorder: a two-sample Mendelian randomisation study
Source: Gen Psychiatr. 2022 Sep 27;35(5):e100807. doi: 10.1136/gpsych-2022-100807 (PMC9516288; doi:10.1136/gpsych-2022-100807)
Supplement: Supplementary data [file gpsych-2022-100807supp003.pdf]

Table S2. Calculation of F-statistic.

$$F = \frac{N-k-1}{k}\frac{R^2}{1-R^2}$$

Left HGS: F-statistic = 1122.09

Right HGS: F-statistic = 1005.31

N: Sample size

k: The number of instrumental variants

R^2: The proportion of variance in the risk factor explained by the IVs
