# Supplementary material for: Handgrip strength and the risk of major depressive disorder: a two-sample Mendelian randomisation study
Source: Gen Psychiatr. 2022 Sep 27;35(5):e100807. doi: 10.1136/gpsych-2022-100807 (PMC9516288; doi:10.1136/gpsych-2022-100807)
Supplement: Supplementary data [file gpsych-2022-100807supp004.pdf]

Table S3. Detailed SNPs information about Leave-one-out method

	exposure	outcome	SNP	b	se	p
1	Hand grip strength (right) || id:ukb-b-10215	Major Depressive Disorder || id:ieu-a-1188	rs10278546	-0.161757083	0.091052366	0.075645922
2	Hand grip strength (right) || id:ukb-b-10215	Major Depressive Disorder || id:ieu-a-1188	rs1043515	-0.179267598	0.090489458	0.047581382
3	Hand grip strength (right) || id:ukb-b-10215	Major Depressive Disorder || id:ieu-a-1188	rs1047891	-0.159949782	0.091069059	0.079027467
4	Hand grip strength (right) || id:ukb-b-10215	Major Depressive Disorder || id:ieu-a-1188	rs10483727	-0.162102666	0.091070414	0.075080762
5	Hand grip strength (right) || id:ukb-b-10215	Major Depressive Disorder || id:ieu-a-1188	rs10520770	-0.171692955	0.090840694	0.058752008
6	Hand grip strength (right) || id:ukb-b-10215	Major Depressive Disorder || id:ieu-a-1188	rs10761411	-0.164348776	0.090957525	0.070781947
7	Hand grip strength (right) || id:ukb-b-10215	Major Depressive Disorder || id:ieu-a-1188	rs10770125	-0.167612107	0.090652708	0.064465379
8	Hand grip strength (right) || id:ukb-b-10215	Major Depressive Disorder || id:ieu-a-1188	rs10784502	-0.154658511	0.091053822	0.089405988
9	Hand grip strength (right) || id:ukb-b-10215	Major Depressive Disorder || id:ieu-a-1188	rs10798483	-0.155902158	0.091453688	0.088248234
10	Hand grip strength (right) || id:ukb-b-10215	Major Depressive Disorder || id:ieu-a-1188	rs10799428	-0.162589847	0.091205364	0.074638642
11	Hand grip strength (right) || id:ukb-b-10215	Major Depressive Disorder || id:ieu-a-1188	rs11022513	-0.160300894	0.091084842	0.078423496
12	Hand grip strength (right) || id:ukb-b-10215	Major Depressive Disorder || id:ieu-a-1188	rs11039348	-0.174976377	0.089830848	0.051433767
13	Hand grip strength (right) || id:ukb-b-10215	Major Depressive Disorder || id:ieu-a-1188	rs11243202	-0.169543183	0.09098713	0.062409465
14	Hand grip strength (right) || id:ukb-b-10215	Major Depressive Disorder || id:ieu-a-1188	rs1125	-0.150249081	0.090433176	0.096625586
15	Hand grip strength (right) || id:ukb-b-10215	Major Depressive Disorder || id:ieu-a-1188	rs113315602	-0.160666508	0.090859534	0.077011508
16	Hand grip strength (right) || id:ukb-b-10215	Major Depressive Disorder || id:ieu-a-1188	rs113851275	-0.176169745	0.089263929	0.048429215
17	Hand grip strength (right) || id:ukb-b-10215	Major Depressive Disorder || id:ieu-a-1188	rs114924396	-0.165567367	0.090923255	0.068613345
18	Hand grip strength (right) || id:ukb-b-10215	Major Depressive Disorder || id:ieu-a-1188	rs11642954	-0.164572518	0.091129521	0.07093102
19	Hand grip strength (right) || id:ukb-b-10215	Major Depressive Disorder || id:ieu-a-1188	rs116922558	-0.154228608	0.090651755	0.088880918
20	Hand grip strength (right) || id:ukb-b-10215	Major Depressive Disorder || id:ieu-a-1188	rs11998884	-0.161827238	0.090977406	0.075278637
21	Hand grip strength (right) || id:ukb-b-10215	Major Depressive Disorder || id:ieu-a-1188	rs12052508	-0.158654788	0.091017943	0.081313439
22	Hand grip strength (right) || id:ukb-b-10215	Major Depressive Disorder || id:ieu-a-1188	rs12316046	-0.159204918	0.091625512	0.082288194
23	Hand grip strength (right) || id:ukb-b-10215	Major Depressive Disorder || id:ieu-a-1188	rs12412806	-0.168927973	0.090545609	0.062087942
24	Hand grip strength (right) || id:ukb-b-10215	Major Depressive Disorder || id:ieu-a-1188	rs12522139	-0.165228676	0.090941934	0.069238697
25	Hand grip strength (right) || id:ukb-b-10215	Major Depressive Disorder || id:ieu-a-1188	rs12616285	-0.172103386	0.089982337	0.05579477
26	Hand grip strength (right) || id:ukb-b-10215	Major Depressive Disorder || id:ieu-a-1188	rs12763284	-0.151616569	0.090605783	0.094255413
27	Hand grip strength (right) || id:ukb-b-10215	Major Depressive Disorder || id:ieu-a-1188	rs12790261	-0.158509811	0.091199859	0.082202205
28	Hand grip strength (right) || id:ukb-b-10215	Major Depressive Disorder || id:ieu-a-1188	rs12823922	-0.15490973	0.090883656	0.088290719
29	Hand grip strength (right) || id:ukb-b-10215	Major Depressive Disorder || id:ieu-a-1188	rs12889267	-0.1547806	0.090816635	0.088321335
30	Hand grip strength (right) || id:ukb-b-10215	Major Depressive Disorder || id:ieu-a-1188	rs12899474	-0.156243128	0.090946899	0.085803926
31	Hand grip strength (right) || id:ukb-b-10215	Major Depressive Disorder || id:ieu-a-1188	rs12914702	-0.160476881	0.090984772	0.077769472
32	Hand grip strength (right) || id:ukb-b-10215	Major Depressive Disorder || id:ieu-a-1188	rs13106087	-0.166443482	0.090927685	0.067174625
33	Hand grip strength (right) || id:ukb-b-10215	Major Depressive Disorder || id:ieu-a-1188	rs13107325	-0.173076993	0.090952601	0.057048876
34	Hand grip strength (right) || id:ukb-b-10215	Major Depressive Disorder || id:ieu-a-1188	rs13146142	-0.152692624	0.091203761	0.094092836
35	Hand grip strength (right) || id:ukb-b-10215	Major Depressive Disorder || id:ieu-a-1188	rs13169333	-0.153046377	0.090538264	0.090950407
36	Hand grip strength (right) || id:ukb-b-10215	Major Depressive Disorder || id:ieu-a-1188	rs13355365	-0.163626519	0.090996177	0.072150204

37	Hand grip strength (right) || id:ukb-b-10215	Major Depressive Disorder || id:ieu-a-1188	rs13356200	-0.156939082	0.09095694	0.084451497
38	Hand grip strength (right) || id:ukb-b-10215	Major Depressive Disorder || id:ieu-a-1188	rs143384	-0.155007767	0.092488004	0.093742699
39	Hand grip strength (right) || id:ukb-b-10215	Major Depressive Disorder || id:ieu-a-1188	rs1442883	-0.159995753	0.091089432	0.07900838
40	Hand grip strength (right) || id:ukb-b-10215	Major Depressive Disorder || id:ieu-a-1188	rs1486925	-0.163543344	0.091039648	0.072431413
41	Hand grip strength (right) || id:ukb-b-10215	Major Depressive Disorder || id:ieu-a-1188	rs150330307	-0.166238818	0.091087067	0.067992879
42	Hand grip strength (right) || id:ukb-b-10215	Major Depressive Disorder || id:ieu-a-1188	rs1550115	-0.1451359	0.090603309	0.109181486
43	Hand grip strength (right) || id:ukb-b-10215	Major Depressive Disorder || id:ieu-a-1188	rs1556659	-0.170704665	0.091546878	0.062227755
44	Hand grip strength (right) || id:ukb-b-10215	Major Depressive Disorder || id:ieu-a-1188	rs1641457	-0.163567071	0.091193932	0.072874174
45	Hand grip strength (right) || id:ukb-b-10215	Major Depressive Disorder || id:ieu-a-1188	rs1840753	-0.168429181	0.090600607	0.06302299
46	Hand grip strength (right) || id:ukb-b-10215	Major Depressive Disorder || id:ieu-a-1188	rs1885690	-0.152661176	0.090497179	0.091619429
47	Hand grip strength (right) || id:ukb-b-10215	Major Depressive Disorder || id:ieu-a-1188	rs1892425	-0.155443425	0.090935131	0.087379097
48	Hand grip strength (right) || id:ukb-b-10215	Major Depressive Disorder || id:ieu-a-1188	rs1952256	-0.165942985	0.090960872	0.068101638
49	Hand grip strength (right) || id:ukb-b-10215	Major Depressive Disorder || id:ieu-a-1188	rs2147461	-0.153278789	0.090663558	0.090906946
50	Hand grip strength (right) || id:ukb-b-10215	Major Depressive Disorder || id:ieu-a-1188	rs2165241	-0.166016965	0.091217429	0.068756928
51	Hand grip strength (right) || id:ukb-b-10215	Major Depressive Disorder || id:ieu-a-1188	rs2194411	-0.158631565	0.091062344	0.081506595
52	Hand grip strength (right) || id:ukb-b-10215	Major Depressive Disorder || id:ieu-a-1188	rs2194747	-0.159520515	0.091005431	0.079624655
53	Hand grip strength (right) || id:ukb-b-10215	Major Depressive Disorder || id:ieu-a-1188	rs2208562	-0.156630875	0.091159967	0.085760502
54	Hand grip strength (right) || id:ukb-b-10215	Major Depressive Disorder || id:ieu-a-1188	rs2226685	-0.16744521	0.090794221	0.065149974
55	Hand grip strength (right) || id:ukb-b-10215	Major Depressive Disorder || id:ieu-a-1188	rs2244621	-0.157209496	0.090905738	0.083743211
56	Hand grip strength (right) || id:ukb-b-10215	Major Depressive Disorder || id:ieu-a-1188	rs2273555	-0.158544765	0.091185938	0.082087982
57	Hand grip strength (right) || id:ukb-b-10215	Major Depressive Disorder || id:ieu-a-1188	rs2296316	-0.157770219	0.090950656	0.082797627
58	Hand grip strength (right) || id:ukb-b-10215	Major Depressive Disorder || id:ieu-a-1188	rs2322754	-0.164451555	0.091005892	0.070755585
59	Hand grip strength (right) || id:ukb-b-10215	Major Depressive Disorder || id:ieu-a-1188	rs2341184	-0.168448858	0.09073408	0.063380693
60	Hand grip strength (right) || id:ukb-b-10215	Major Depressive Disorder || id:ieu-a-1188	rs2362972	-0.174830182	0.089632428	0.051113724
61	Hand grip strength (right) || id:ukb-b-10215	Major Depressive Disorder || id:ieu-a-1188	rs2389763	-0.164144349	0.090963386	0.071151376
62	Hand grip strength (right) || id:ukb-b-10215	Major Depressive Disorder || id:ieu-a-1188	rs2431112	-0.124786929	0.085248549	0.143248274
63	Hand grip strength (right) || id:ukb-b-10215	Major Depressive Disorder || id:ieu-a-1188	rs246181	-0.162110324	0.091090486	0.075131207
64	Hand grip strength (right) || id:ukb-b-10215	Major Depressive Disorder || id:ieu-a-1188	rs248831	-0.157014543	0.090922666	0.084185246
65	Hand grip strength (right) || id:ukb-b-10215	Major Depressive Disorder || id:ieu-a-1188	rs2587505	-0.16484151	0.090949008	0.069915028
66	Hand grip strength (right) || id:ukb-b-10215	Major Depressive Disorder || id:ieu-a-1188	rs2631360	-0.166699289	0.091100347	0.06727349
67	Hand grip strength (right) || id:ukb-b-10215	Major Depressive Disorder || id:ieu-a-1188	rs2717351	-0.176525618	0.089991776	0.049811985
68	Hand grip strength (right) || id:ukb-b-10215	Major Depressive Disorder || id:ieu-a-1188	rs2854152	-0.160644206	0.091080278	0.077771554
69	Hand grip strength (right) || id:ukb-b-10215	Major Depressive Disorder || id:ieu-a-1188	rs2894602	-0.159867225	0.091045787	0.079105621
70	Hand grip strength (right) || id:ukb-b-10215	Major Depressive Disorder || id:ieu-a-1188	rs3118914	-0.168661671	0.091554583	0.065446191
71	Hand grip strength (right) || id:ukb-b-10215	Major Depressive Disorder || id:ieu-a-1188	rs34030812	-0.151143169	0.09039284	0.094510489
72	Hand grip strength (right) || id:ukb-b-10215	Major Depressive Disorder || id:ieu-a-1188	rs34845616	-0.16159665	0.091002417	0.075775747
73	Hand grip strength (right) || id:ukb-b-10215	Major Depressive Disorder || id:ieu-a-1188	rs35175534	-0.162481313	0.090650624	0.073070444
74	Hand grip strength (right) || id:ukb-b-10215	Major Depressive Disorder || id:ieu-a-1188	rs35304341	-0.160345312	0.091014834	0.078111679

75	Hand grip strength (right) || id:ukb-b-10215	Major Depressive Disorder || id:ieu-a-1188	rs35457492	-0.16818455	0.090661677	0.063584982
76	Hand grip strength (right) || id:ukb-b-10215	Major Depressive Disorder || id:ieu-a-1188	rs35701422	-0.158085311	0.090985605	0.082303176
77	Hand grip strength (right) || id:ukb-b-10215	Major Depressive Disorder || id:ieu-a-1188	rs35833641	-0.163839017	0.091009167	0.071821583
78	Hand grip strength (right) || id:ukb-b-10215	Major Depressive Disorder || id:ieu-a-1188	rs36065733	-0.165226452	0.090969691	0.069327395
79	Hand grip strength (right) || id:ukb-b-10215	Major Depressive Disorder || id:ieu-a-1188	rs3771498	-0.152127901	0.091275061	0.095574604
80	Hand grip strength (right) || id:ukb-b-10215	Major Depressive Disorder || id:ieu-a-1188	rs3848369	-0.159238592	0.091033821	0.080252218
81	Hand grip strength (right) || id:ukb-b-10215	Major Depressive Disorder || id:ieu-a-1188	rs4121165	-0.15497611	0.090916903	0.088270782
82	Hand grip strength (right) || id:ukb-b-10215	Major Depressive Disorder || id:ieu-a-1188	rs4369779	-0.173742167	0.091014498	0.056268485
83	Hand grip strength (right) || id:ukb-b-10215	Major Depressive Disorder || id:ieu-a-1188	rs4549685	-0.160361771	0.091094761	0.078342701
84	Hand grip strength (right) || id:ukb-b-10215	Major Depressive Disorder || id:ieu-a-1188	rs4553566	-0.166815855	0.090903911	0.066493991
85	Hand grip strength (right) || id:ukb-b-10215	Major Depressive Disorder || id:ieu-a-1188	rs4737446	-0.151627148	0.090558465	0.094060571
86	Hand grip strength (right) || id:ukb-b-10215	Major Depressive Disorder || id:ieu-a-1188	rs4751671	-0.163713271	0.09098405	0.071961385
87	Hand grip strength (right) || id:ukb-b-10215	Major Depressive Disorder || id:ieu-a-1188	rs4752689	-0.155497435	0.090846021	0.086959477
88	Hand grip strength (right) || id:ukb-b-10215	Major Depressive Disorder || id:ieu-a-1188	rs4768725	-0.156778471	0.090908245	0.084603328
89	Hand grip strength (right) || id:ukb-b-10215	Major Depressive Disorder || id:ieu-a-1188	rs4784329	-0.17357972	0.090914877	0.056229151
90	Hand grip strength (right) || id:ukb-b-10215	Major Depressive Disorder || id:ieu-a-1188	rs4785574	-0.151105015	0.090662003	0.095577025
91	Hand grip strength (right) || id:ukb-b-10215	Major Depressive Disorder || id:ieu-a-1188	rs4793658	-0.156804329	0.090776631	0.084102222
92	Hand grip strength (right) || id:ukb-b-10215	Major Depressive Disorder || id:ieu-a-1188	rs4927015	-0.164186147	0.091300509	0.072128713
93	Hand grip strength (right) || id:ukb-b-10215	Major Depressive Disorder || id:ieu-a-1188	rs56074046	-0.146830591	0.089544184	0.101055961
94	Hand grip strength (right) || id:ukb-b-10215	Major Depressive Disorder || id:ieu-a-1188	rs56144131	-0.168400912	0.090764309	0.063544138
95	Hand grip strength (right) || id:ukb-b-10215	Major Depressive Disorder || id:ieu-a-1188	rs56365901	-0.160714206	0.091150104	0.077869829
96	Hand grip strength (right) || id:ukb-b-10215	Major Depressive Disorder || id:ieu-a-1188	rs58670122	-0.156787219	0.090936422	0.08468238
97	Hand grip strength (right) || id:ukb-b-10215	Major Depressive Disorder || id:ieu-a-1188	rs600038	-0.164306069	0.090979698	0.070923948
98	Hand grip strength (right) || id:ukb-b-10215	Major Depressive Disorder || id:ieu-a-1188	rs6006984	-0.166837067	0.090771693	0.066064522
99	Hand grip strength (right) || id:ukb-b-10215	Major Depressive Disorder || id:ieu-a-1188	rs61389091	-0.162463107	0.091064653	0.074416946
100	Hand grip strength (right) || id:ukb-b-10215	Major Depressive Disorder || id:ieu-a-1188	rs62037412	-0.166232364	0.090891282	0.067412449
101	Hand grip strength (right) || id:ukb-b-10215	Major Depressive Disorder || id:ieu-a-1188	rs62234790	-0.156146726	0.090947251	0.085998669
102	Hand grip strength (right) || id:ukb-b-10215	Major Depressive Disorder || id:ieu-a-1188	rs62509875	-0.162182237	0.091128186	0.075122547
103	Hand grip strength (right) || id:ukb-b-10215	Major Depressive Disorder || id:ieu-a-1188	rs635538	-0.137133265	0.089251463	0.124420213
104	Hand grip strength (right) || id:ukb-b-10215	Major Depressive Disorder || id:ieu-a-1188	rs645144	-0.161338396	0.091031987	0.076340385
105	Hand grip strength (right) || id:ukb-b-10215	Major Depressive Disorder || id:ieu-a-1188	rs6473015	-0.163400971	0.09103502	0.072665758
106	Hand grip strength (right) || id:ukb-b-10215	Major Depressive Disorder || id:ieu-a-1188	rs6693965	-0.170097891	0.090858273	0.061189546
107	Hand grip strength (right) || id:ukb-b-10215	Major Depressive Disorder || id:ieu-a-1188	rs6711390	-0.155867369	0.091128326	0.08718893
108	Hand grip strength (right) || id:ukb-b-10215	Major Depressive Disorder || id:ieu-a-1188	rs6715064	-0.156098652	0.090863751	0.0858072
109	Hand grip strength (right) || id:ukb-b-10215	Major Depressive Disorder || id:ieu-a-1188	rs6792762	-0.156809525	0.090950857	0.084687507
110	Hand grip strength (right) || id:ukb-b-10215	Major Depressive Disorder || id:ieu-a-1188	rs6882168	-0.156148696	0.090876202	0.085749696
111	Hand grip strength (right) || id:ukb-b-10215	Major Depressive Disorder || id:ieu-a-1188	rs6962338	-0.156227072	0.090844863	0.085484665
112	Hand grip strength (right) || id:ukb-b-10215	Major Depressive Disorder || id:ieu-a-1188	rs6977081	-0.168692873	0.09109443	0.064047914

113	Hand grip strength (right) || id:ukb-b-10215	Major Depressive Disorder || id:ieu-a-1188	rs7034200	-0.170854228	0.09039072	0.058734635
114	Hand grip strength (right) || id:ukb-b-10215	Major Depressive Disorder || id:ieu-a-1188	rs71298370	-0.166067764	0.090860995	0.067592733
115	Hand grip strength (right) || id:ukb-b-10215	Major Depressive Disorder || id:ieu-a-1188	rs7148603	-0.173566583	0.089945548	0.053645868
116	Hand grip strength (right) || id:ukb-b-10215	Major Depressive Disorder || id:ieu-a-1188	rs7196917	-0.153115832	0.090872753	0.091998671
117	Hand grip strength (right) || id:ukb-b-10215	Major Depressive Disorder || id:ieu-a-1188	rs7206195	-0.154074478	0.090709125	0.089402978
118	Hand grip strength (right) || id:ukb-b-10215	Major Depressive Disorder || id:ieu-a-1188	rs721101	-0.158917535	0.091020225	0.080818165
119	Hand grip strength (right) || id:ukb-b-10215	Major Depressive Disorder || id:ieu-a-1188	rs7214252	-0.149061257	0.089935627	0.097434253
120	Hand grip strength (right) || id:ukb-b-10215	Major Depressive Disorder || id:ieu-a-1188	rs7249	-0.173233719	0.089673032	0.053379575
121	Hand grip strength (right) || id:ukb-b-10215	Major Depressive Disorder || id:ieu-a-1188	rs7266065	-0.170784939	0.090531454	0.059231547
122	Hand grip strength (right) || id:ukb-b-10215	Major Depressive Disorder || id:ieu-a-1188	rs7301953	-0.150947407	0.09072498	0.0961542
123	Hand grip strength (right) || id:ukb-b-10215	Major Depressive Disorder || id:ieu-a-1188	rs7451021	-0.173731931	0.091141596	0.056627363
124	Hand grip strength (right) || id:ukb-b-10215	Major Depressive Disorder || id:ieu-a-1188	rs75457267	-0.165394238	0.090869131	0.068738193
125	Hand grip strength (right) || id:ukb-b-10215	Major Depressive Disorder || id:ieu-a-1188	rs7549184	-0.162164095	0.091063218	0.074947432
126	Hand grip strength (right) || id:ukb-b-10215	Major Depressive Disorder || id:ieu-a-1188	rs7565148	-0.157127036	0.09109872	0.084563594
127	Hand grip strength (right) || id:ukb-b-10215	Major Depressive Disorder || id:ieu-a-1188	rs7657558	-0.15261087	0.090698097	0.092447741
128	Hand grip strength (right) || id:ukb-b-10215	Major Depressive Disorder || id:ieu-a-1188	rs76749769	-0.166239922	0.090776936	0.067055582
129	Hand grip strength (right) || id:ukb-b-10215	Major Depressive Disorder || id:ieu-a-1188	rs76895963	-0.16398744	0.090935595	0.071335449
130	Hand grip strength (right) || id:ukb-b-10215	Major Depressive Disorder || id:ieu-a-1188	rs7790322	-0.156676443	0.090917124	0.08483635
131	Hand grip strength (right) || id:ukb-b-10215	Major Depressive Disorder || id:ieu-a-1188	rs7871404	-0.157900874	0.091048328	0.082873129
132	Hand grip strength (right) || id:ukb-b-10215	Major Depressive Disorder || id:ieu-a-1188	rs7963801	-0.170228728	0.090850801	0.060969384
133	Hand grip strength (right) || id:ukb-b-10215	Major Depressive Disorder || id:ieu-a-1188	rs79723785	-0.162797774	0.090827474	0.073071363
134	Hand grip strength (right) || id:ukb-b-10215	Major Depressive Disorder || id:ieu-a-1188	rs8055199	-0.165553694	0.090844389	0.068396184
135	Hand grip strength (right) || id:ukb-b-10215	Major Depressive Disorder || id:ieu-a-1188	rs823130	-0.15098472	0.09075204	0.096171182
136	Hand grip strength (right) || id:ukb-b-10215	Major Depressive Disorder || id:ieu-a-1188	rs852520	-0.160493355	0.09104044	0.07792076
137	Hand grip strength (right) || id:ukb-b-10215	Major Depressive Disorder || id:ieu-a-1188	rs911642	-0.158115255	0.090975617	0.082211525
138	Hand grip strength (right) || id:ukb-b-10215	Major Depressive Disorder || id:ieu-a-1188	rs9322822	-0.145320552	0.089913032	0.106043604
139	Hand grip strength (right) || id:ukb-b-10215	Major Depressive Disorder || id:ieu-a-1188	rs935728	-0.164963717	0.09098848	0.069829104
140	Hand grip strength (right) || id:ukb-b-10215	Major Depressive Disorder || id:ieu-a-1188	rs9388051	-0.157220688	0.090921912	0.083776225
141	Hand grip strength (right) || id:ukb-b-10215	Major Depressive Disorder || id:ieu-a-1188	rs9396861	-0.158445565	0.091033475	0.081767787
142	Hand grip strength (right) || id:ukb-b-10215	Major Depressive Disorder || id:ieu-a-1188	rs9652468	-0.166129868	0.091086992	0.068173331
143	Hand grip strength (right) || id:ukb-b-10215	Major Depressive Disorder || id:ieu-a-1188	rs9757079	-0.157734488	0.091024528	0.083117186
144	Hand grip strength (right) || id:ukb-b-10215	Major Depressive Disorder || id:ieu-a-1188	rs9853018	-0.156727007	0.091068823	0.085255682
145	Hand grip strength (right) || id:ukb-b-10215	Major Depressive Disorder || id:ieu-a-1188	All	-0.161072931	0.090504806	0.075122307
146	Hand grip strength (left) || id:ukb-b-7478	Major Depressive Disorder || id:ieu-a-1188	rs10097417	-0.248141794	0.094118437	0.008377005
147	Hand grip strength (left) || id:ukb-b-7478	Major Depressive Disorder || id:ieu-a-1188	rs10176878	-0.248088571	0.094124688	0.008395307
148	Hand grip strength (left) || id:ukb-b-7478	Major Depressive Disorder || id:ieu-a-1188	rs10403906	-0.240538618	0.09392867	0.0104412
149	Hand grip strength (left) || id:ukb-b-7478	Major Depressive Disorder || id:ieu-a-1188	rs1044299	-0.241668271	0.094420435	0.010482551
150	Hand grip strength (left) || id:ukb-b-7478	Major Depressive Disorder || id:ieu-a-1188	rs10786706	-0.236906008	0.093636647	0.011404416

151	Hand grip strength (left) || id:ukb-b-7478	Major Depressive Disorder || id:ieu-a-1188	rs10821939	-0.243433027	0.094007498	0.009611361
152	Hand grip strength (left) || id:ukb-b-7478	Major Depressive Disorder || id:ieu-a-1188	rs10831903	-0.246936774	0.094063371	0.008659372
153	Hand grip strength (left) || id:ukb-b-7478	Major Depressive Disorder || id:ieu-a-1188	rs10934857	-0.243214088	0.093892046	0.009587594
154	Hand grip strength (left) || id:ukb-b-7478	Major Depressive Disorder || id:ieu-a-1188	rs10988217	-0.254590812	0.093648849	0.006556507
155	Hand grip strength (left) || id:ukb-b-7478	Major Depressive Disorder || id:ieu-a-1188	rs11002322	-0.246047029	0.094100304	0.008929762
156	Hand grip strength (left) || id:ukb-b-7478	Major Depressive Disorder || id:ieu-a-1188	rs11076004	-0.260841131	0.093452665	0.005251987
157	Hand grip strength (left) || id:ukb-b-7478	Major Depressive Disorder || id:ieu-a-1188	rs11111267	-0.248454508	0.094005224	0.008217839
158	Hand grip strength (left) || id:ukb-b-7478	Major Depressive Disorder || id:ieu-a-1188	rs11121542	-0.256582158	0.093713675	0.006182588
159	Hand grip strength (left) || id:ukb-b-7478	Major Depressive Disorder || id:ieu-a-1188	rs11125803	-0.233138616	0.093692215	0.012833893
160	Hand grip strength (left) || id:ukb-b-7478	Major Depressive Disorder || id:ieu-a-1188	rs11168357	-0.250180951	0.093913732	0.007723035
161	Hand grip strength (left) || id:ukb-b-7478	Major Depressive Disorder || id:ieu-a-1188	rs11204664	-0.245335127	0.09399562	0.009052376
162	Hand grip strength (left) || id:ukb-b-7478	Major Depressive Disorder || id:ieu-a-1188	rs11243202	-0.255000854	0.093741663	0.006523237
163	Hand grip strength (left) || id:ukb-b-7478	Major Depressive Disorder || id:ieu-a-1188	rs112485536	-0.248669091	0.094016683	0.008170385
164	Hand grip strength (left) || id:ukb-b-7478	Major Depressive Disorder || id:ieu-a-1188	rs113315602	-0.246394068	0.093790669	0.008612519
165	Hand grip strength (left) || id:ukb-b-7478	Major Depressive Disorder || id:ieu-a-1188	rs113434679	-0.246338455	0.094144255	0.00888087
166	Hand grip strength (left) || id:ukb-b-7478	Major Depressive Disorder || id:ieu-a-1188	rs113918482	-0.236230974	0.093186029	0.01124322
167	Hand grip strength (left) || id:ukb-b-7478	Major Depressive Disorder || id:ieu-a-1188	rs116409670	-0.252304041	0.093773186	0.007132843
168	Hand grip strength (left) || id:ukb-b-7478	Major Depressive Disorder || id:ieu-a-1188	rs11642954	-0.250644371	0.093999559	0.007665883
169	Hand grip strength (left) || id:ukb-b-7478	Major Depressive Disorder || id:ieu-a-1188	rs116922558	-0.240321963	0.093520294	0.010177763
170	Hand grip strength (left) || id:ukb-b-7478	Major Depressive Disorder || id:ieu-a-1188	rs12316046	-0.246732494	0.09483945	0.009279519
171	Hand grip strength (left) || id:ukb-b-7478	Major Depressive Disorder || id:ieu-a-1188	rs12473732	-0.239882997	0.093999511	0.01071188
172	Hand grip strength (left) || id:ukb-b-7478	Major Depressive Disorder || id:ieu-a-1188	rs12528131	-0.237811245	0.093500641	0.010977372
173	Hand grip strength (left) || id:ukb-b-7478	Major Depressive Disorder || id:ieu-a-1188	rs12533765	-0.238405938	0.093469065	0.010752532
174	Hand grip strength (left) || id:ukb-b-7478	Major Depressive Disorder || id:ieu-a-1188	rs12673062	-0.250215798	0.09396203	0.007745988
175	Hand grip strength (left) || id:ukb-b-7478	Major Depressive Disorder || id:ieu-a-1188	rs12790261	-0.244721145	0.094166668	0.009354793
176	Hand grip strength (left) || id:ukb-b-7478	Major Depressive Disorder || id:ieu-a-1188	rs12889267	-0.239716451	0.09387745	0.010664503
177	Hand grip strength (left) || id:ukb-b-7478	Major Depressive Disorder || id:ieu-a-1188	rs12906830	-0.246716947	0.09418454	0.008805728
178	Hand grip strength (left) || id:ukb-b-7478	Major Depressive Disorder || id:ieu-a-1188	rs12914702	-0.246431058	0.093956147	0.008720262
179	Hand grip strength (left) || id:ukb-b-7478	Major Depressive Disorder || id:ieu-a-1188	rs13091492	-0.25200684	0.093795699	0.007214806
180	Hand grip strength (left) || id:ukb-b-7478	Major Depressive Disorder || id:ieu-a-1188	rs13106087	-0.252481859	0.093776647	0.007094522
181	Hand grip strength (left) || id:ukb-b-7478	Major Depressive Disorder || id:ieu-a-1188	rs13107325	-0.260422082	0.093734553	0.005464527
182	Hand grip strength (left) || id:ukb-b-7478	Major Depressive Disorder || id:ieu-a-1188	rs13146142	-0.23878417	0.094206275	0.011254528
183	Hand grip strength (left) || id:ukb-b-7478	Major Depressive Disorder || id:ieu-a-1188	rs13227429	-0.245946018	0.093969722	0.0088631
184	Hand grip strength (left) || id:ukb-b-7478	Major Depressive Disorder || id:ieu-a-1188	rs13337177	-0.244916592	0.094053172	0.009213666
185	Hand grip strength (left) || id:ukb-b-7478	Major Depressive Disorder || id:ieu-a-1188	rs13356200	-0.242794091	0.093907781	0.009725132
186	Hand grip strength (left) || id:ukb-b-7478	Major Depressive Disorder || id:ieu-a-1188	rs143002906	-0.245461018	0.094012589	0.009029432
187	Hand grip strength (left) || id:ukb-b-7478	Major Depressive Disorder || id:ieu-a-1188	rs143384	-0.243028574	0.095352203	0.010811092
188	Hand grip strength (left) || id:ukb-b-7478	Major Depressive Disorder || id:ieu-a-1188	rs1434095	-0.250461635	0.093978899	0.007696915

189	Hand grip strength (left) || id:ukb-b-7478	Major Depressive Disorder || id:ieu-a-1188	rs1486925	-0.250529678	0.094040464	0.00772032
190	Hand grip strength (left) || id:ukb-b-7478	Major Depressive Disorder || id:ieu-a-1188	rs150330307	-0.252805953	0.093972224	0.007140493
191	Hand grip strength (left) || id:ukb-b-7478	Major Depressive Disorder || id:ieu-a-1188	rs1551042	-0.242521474	0.094103712	0.00996123
192	Hand grip strength (left) || id:ukb-b-7478	Major Depressive Disorder || id:ieu-a-1188	rs1556659	-0.258089137	0.094372603	0.006242031
193	Hand grip strength (left) || id:ukb-b-7478	Major Depressive Disorder || id:ieu-a-1188	rs1641457	-0.249887182	0.09409861	0.007917017
194	Hand grip strength (left) || id:ukb-b-7478	Major Depressive Disorder || id:ieu-a-1188	rs16870531	-0.247494771	0.094100033	0.008535368
195	Hand grip strength (left) || id:ukb-b-7478	Major Depressive Disorder || id:ieu-a-1188	rs17282763	-0.228094298	0.091290487	0.012470081
196	Hand grip strength (left) || id:ukb-b-7478	Major Depressive Disorder || id:ieu-a-1188	rs17466480	-0.254504809	0.094011048	0.00678575
197	Hand grip strength (left) || id:ukb-b-7478	Major Depressive Disorder || id:ieu-a-1188	rs17630248	-0.238974208	0.093638214	0.010707538
198	Hand grip strength (left) || id:ukb-b-7478	Major Depressive Disorder || id:ieu-a-1188	rs181766	-0.245994356	0.094053323	0.008910278
199	Hand grip strength (left) || id:ukb-b-7478	Major Depressive Disorder || id:ieu-a-1188	rs1884447	-0.243479422	0.093937036	0.009543564
200	Hand grip strength (left) || id:ukb-b-7478	Major Depressive Disorder || id:ieu-a-1188	rs1981612	-0.25350681	0.093757004	0.006853697
201	Hand grip strength (left) || id:ukb-b-7478	Major Depressive Disorder || id:ieu-a-1188	rs2038760	-0.247069546	0.094039072	0.008606356
202	Hand grip strength (left) || id:ukb-b-7478	Major Depressive Disorder || id:ieu-a-1188	rs217181	-0.240609305	0.093879155	0.01037815
203	Hand grip strength (left) || id:ukb-b-7478	Major Depressive Disorder || id:ieu-a-1188	rs2359239	-0.262354465	0.0923544	0.004500956
204	Hand grip strength (left) || id:ukb-b-7478	Major Depressive Disorder || id:ieu-a-1188	rs2431112	-0.212592679	0.087060765	0.014610659
205	Hand grip strength (left) || id:ukb-b-7478	Major Depressive Disorder || id:ieu-a-1188	rs2532111	-0.250134636	0.094031271	0.007811239
206	Hand grip strength (left) || id:ukb-b-7478	Major Depressive Disorder || id:ieu-a-1188	rs2587505	-0.251243542	0.09385488	0.007429874
207	Hand grip strength (left) || id:ukb-b-7478	Major Depressive Disorder || id:ieu-a-1188	rs2631360	-0.253640202	0.09400459	0.006972219
208	Hand grip strength (left) || id:ukb-b-7478	Major Depressive Disorder || id:ieu-a-1188	rs2800789	-0.257888078	0.093032749	0.005571065
209	Hand grip strength (left) || id:ukb-b-7478	Major Depressive Disorder || id:ieu-a-1188	rs28542042	-0.248080997	0.094112632	0.008388947
210	Hand grip strength (left) || id:ukb-b-7478	Major Depressive Disorder || id:ieu-a-1188	rs2871960	-0.242586636	0.094254707	0.01006073
211	Hand grip strength (left) || id:ukb-b-7478	Major Depressive Disorder || id:ieu-a-1188	rs2974438	-0.245101271	0.093978153	0.009105526
212	Hand grip strength (left) || id:ukb-b-7478	Major Depressive Disorder || id:ieu-a-1188	rs3118903	-0.254362086	0.094400566	0.007049486
213	Hand grip strength (left) || id:ukb-b-7478	Major Depressive Disorder || id:ieu-a-1188	rs34030812	-0.23520051	0.093399591	0.011795037
214	Hand grip strength (left) || id:ukb-b-7478	Major Depressive Disorder || id:ieu-a-1188	rs34722008	-0.237854911	0.093374631	0.010855435
215	Hand grip strength (left) || id:ukb-b-7478	Major Depressive Disorder || id:ieu-a-1188	rs34845616	-0.247934813	0.094006016	0.008353548
216	Hand grip strength (left) || id:ukb-b-7478	Major Depressive Disorder || id:ieu-a-1188	rs35175534	-0.248091081	0.093539774	0.007995677
217	Hand grip strength (left) || id:ukb-b-7478	Major Depressive Disorder || id:ieu-a-1188	rs35236379	-0.241054741	0.093799589	0.010173045
218	Hand grip strength (left) || id:ukb-b-7478	Major Depressive Disorder || id:ieu-a-1188	rs3814877	-0.239665476	0.093914603	0.010712155
219	Hand grip strength (left) || id:ukb-b-7478	Major Depressive Disorder || id:ieu-a-1188	rs3819121	-0.240762362	0.094176071	0.010572708
220	Hand grip strength (left) || id:ukb-b-7478	Major Depressive Disorder || id:ieu-a-1188	rs4121165	-0.240804396	0.093864812	0.01030455
221	Hand grip strength (left) || id:ukb-b-7478	Major Depressive Disorder || id:ieu-a-1188	rs41271299	-0.249602235	0.093936407	0.007880658
222	Hand grip strength (left) || id:ukb-b-7478	Major Depressive Disorder || id:ieu-a-1188	rs4308051	-0.260565376	0.093793713	0.005468306
223	Hand grip strength (left) || id:ukb-b-7478	Major Depressive Disorder || id:ieu-a-1188	rs4335354	-0.261995148	0.092398601	0.00457557
224	Hand grip strength (left) || id:ukb-b-7478	Major Depressive Disorder || id:ieu-a-1188	rs4498020	-0.243586632	0.094033276	0.009585608
225	Hand grip strength (left) || id:ukb-b-7478	Major Depressive Disorder || id:ieu-a-1188	rs4621706	-0.241905966	0.094177387	0.010210421
226	Hand grip strength (left) || id:ukb-b-7478	Major Depressive Disorder || id:ieu-a-1188	rs4677601	-0.248372653	0.094054281	0.008272582

227	Hand grip strength (left) || id:ukb-b-7478	Major Depressive Disorder || id:ieu-a-1188	rs4737446	-0.236702035	0.09351195	0.011365712
228	Hand grip strength (left) || id:ukb-b-7478	Major Depressive Disorder || id:ieu-a-1188	rs4739739	-0.25785141	0.093028762	0.005575779
229	Hand grip strength (left) || id:ukb-b-7478	Major Depressive Disorder || id:ieu-a-1188	rs4930236	-0.246615895	0.094017903	0.00871404
230	Hand grip strength (left) || id:ukb-b-7478	Major Depressive Disorder || id:ieu-a-1188	rs55681913	-0.251490458	0.093831109	0.007356806
231	Hand grip strength (left) || id:ukb-b-7478	Major Depressive Disorder || id:ieu-a-1188	rs56060323	-0.246632951	0.094023107	0.008713114
232	Hand grip strength (left) || id:ukb-b-7478	Major Depressive Disorder || id:ieu-a-1188	rs56338231	-0.251117508	0.093993621	0.007548064
233	Hand grip strength (left) || id:ukb-b-7478	Major Depressive Disorder || id:ieu-a-1188	rs58670122	-0.242797939	0.093849675	0.009678916
234	Hand grip strength (left) || id:ukb-b-7478	Major Depressive Disorder || id:ieu-a-1188	rs59116179	-0.251010503	0.093869349	0.007494325
235	Hand grip strength (left) || id:ukb-b-7478	Major Depressive Disorder || id:ieu-a-1188	rs6006984	-0.253249674	0.093623354	0.006830768
236	Hand grip strength (left) || id:ukb-b-7478	Major Depressive Disorder || id:ieu-a-1188	rs61389091	-0.24955502	0.09413583	0.008025148
237	Hand grip strength (left) || id:ukb-b-7478	Major Depressive Disorder || id:ieu-a-1188	rs61818100	-0.238545083	0.093609739	0.010825137
238	Hand grip strength (left) || id:ukb-b-7478	Major Depressive Disorder || id:ieu-a-1188	rs62081464	-0.228663262	0.091580325	0.012529837
239	Hand grip strength (left) || id:ukb-b-7478	Major Depressive Disorder || id:ieu-a-1188	rs62253653	-0.248926355	0.094082948	0.008149365
240	Hand grip strength (left) || id:ukb-b-7478	Major Depressive Disorder || id:ieu-a-1188	rs635538	-0.221475585	0.092000267	0.016069364
241	Hand grip strength (left) || id:ukb-b-7478	Major Depressive Disorder || id:ieu-a-1188	rs6433478	-0.247525805	0.09405177	0.00849329
242	Hand grip strength (left) || id:ukb-b-7478	Major Depressive Disorder || id:ieu-a-1188	rs6680160	-0.261478657	0.092906523	0.004886423
243	Hand grip strength (left) || id:ukb-b-7478	Major Depressive Disorder || id:ieu-a-1188	rs6802071	-0.24262693	0.093974568	0.009827625
244	Hand grip strength (left) || id:ukb-b-7478	Major Depressive Disorder || id:ieu-a-1188	rs6882168	-0.241646689	0.093867976	0.010043584
245	Hand grip strength (left) || id:ukb-b-7478	Major Depressive Disorder || id:ieu-a-1188	rs6962338	-0.241604809	0.093843886	0.010037357
246	Hand grip strength (left) || id:ukb-b-7478	Major Depressive Disorder || id:ieu-a-1188	rs6977081	-0.258176191	0.094140946	0.006098401
247	Hand grip strength (left) || id:ukb-b-7478	Major Depressive Disorder || id:ieu-a-1188	rs7026798	-0.246757233	0.094010812	0.008670623
248	Hand grip strength (left) || id:ukb-b-7478	Major Depressive Disorder || id:ieu-a-1188	rs7124681	-0.259085715	0.093663216	0.00567239
249	Hand grip strength (left) || id:ukb-b-7478	Major Depressive Disorder || id:ieu-a-1188	rs71298370	-0.252014264	0.093712729	0.007161893
250	Hand grip strength (left) || id:ukb-b-7478	Major Depressive Disorder || id:ieu-a-1188	rs7148603	-0.261618757	0.09259056	0.004720031
251	Hand grip strength (left) || id:ukb-b-7478	Major Depressive Disorder || id:ieu-a-1188	rs7176095	-0.253735552	0.093554998	0.006684755
252	Hand grip strength (left) || id:ukb-b-7478	Major Depressive Disorder || id:ieu-a-1188	rs7196917	-0.23800123	0.093974208	0.011321268
253	Hand grip strength (left) || id:ukb-b-7478	Major Depressive Disorder || id:ieu-a-1188	rs7197751	-0.248145986	0.094039997	0.008321768
254	Hand grip strength (left) || id:ukb-b-7478	Major Depressive Disorder || id:ieu-a-1188	rs723588	-0.252991072	0.093817939	0.007004773
255	Hand grip strength (left) || id:ukb-b-7478	Major Depressive Disorder || id:ieu-a-1188	rs7516571	-0.243638084	0.093950599	0.009507159
256	Hand grip strength (left) || id:ukb-b-7478	Major Depressive Disorder || id:ieu-a-1188	rs75497896	-0.253035608	0.093846258	0.007011911
257	Hand grip strength (left) || id:ukb-b-7478	Major Depressive Disorder || id:ieu-a-1188	rs755547	-0.261633069	0.093676186	0.005222948
258	Hand grip strength (left) || id:ukb-b-7478	Major Depressive Disorder || id:ieu-a-1188	rs7571789	-0.239330951	0.094209048	0.011071807
259	Hand grip strength (left) || id:ukb-b-7478	Major Depressive Disorder || id:ieu-a-1188	rs76895963	-0.250377918	0.093860631	0.007640654
260	Hand grip strength (left) || id:ukb-b-7478	Major Depressive Disorder || id:ieu-a-1188	rs772014	-0.244735357	0.094158589	0.009344608
261	Hand grip strength (left) || id:ukb-b-7478	Major Depressive Disorder || id:ieu-a-1188	rs7856625	-0.242140158	0.094114403	0.010087136
262	Hand grip strength (left) || id:ukb-b-7478	Major Depressive Disorder || id:ieu-a-1188	rs7963801	-0.256767351	0.093646133	0.006108688
263	Hand grip strength (left) || id:ukb-b-7478	Major Depressive Disorder || id:ieu-a-1188	rs7970350	-0.239424761	0.093878733	0.010761109
264	Hand grip strength (left) || id:ukb-b-7478	Major Depressive Disorder || id:ieu-a-1188	rs8101782	-0.256258311	0.093246655	0.005992765

265	Hand grip strength (left) || id:ukb-b-7478	Major Depressive Disorder || id:ieu-a-1188	rs8108461	-0.249370222	0.093995264	0.007977789
266	Hand grip strength (left) || id:ukb-b-7478	Major Depressive Disorder || id:ieu-a-1188	rs821100	-0.256527687	0.093438383	0.006043334
267	Hand grip strength (left) || id:ukb-b-7478	Major Depressive Disorder || id:ieu-a-1188	rs823130	-0.237042916	0.093639999	0.011359914
268	Hand grip strength (left) || id:ukb-b-7478	Major Depressive Disorder || id:ieu-a-1188	rs9371201	-0.236544877	0.093372402	0.011297842
269	Hand grip strength (left) || id:ukb-b-7478	Major Depressive Disorder || id:ieu-a-1188	rs9371881	-0.248637244	0.094045658	0.008198279
270	Hand grip strength (left) || id:ukb-b-7478	Major Depressive Disorder || id:ieu-a-1188	rs9388769	-0.257269226	0.094099503	0.006256765
271	Hand grip strength (left) || id:ukb-b-7478	Major Depressive Disorder || id:ieu-a-1188	rs9611273	-0.240617754	0.093822822	0.010329562
272	Hand grip strength (left) || id:ukb-b-7478	Major Depressive Disorder || id:ieu-a-1188	rs9866627	-0.248079894	0.094041713	0.008340219
273	Hand grip strength (left) || id:ukb-b-7478	Major Depressive Disorder || id:ieu-a-1188	rs9944324	-0.249234721	0.093984852	0.008004959
274	Hand grip strength (left) || id:ukb-b-7478	Major Depressive Disorder || id:ieu-a-1188	rs999493	-0.232327392	0.093641482	0.013100308
275	Hand grip strength (left) || id:ukb-b-7478	Major Depressive Disorder || id:ieu-a-1188	All	-0.246735109	0.09339371	0.008244691
