# Supplementary material for: Handgrip strength and the risk of major depressive disorder: a two-sample Mendelian randomisation study
Source: Gen Psychiatr. 2022 Sep 27;35(5):e100807. doi: 10.1136/gpsych-2022-100807 (PMC9516288; doi:10.1136/gpsych-2022-100807)
Supplement: Supplementary data [file gpsych-2022-100807supp005.pdf]

Table S4. Heterogeneity test for Mendelian Randomization

	Method	Q	Q_df	Q_pval
Left	MR Egger	221.8	127	3.93E-07
	Inverse variance weighted	222.7	128	4.37E-07
Right	MR Egger	259.5	142	6.62E-09
	Inverse variance weighted	259.8	143	8.33E-09
