# Supplementary material for: Handgrip strength and the risk of major depressive disorder: a two-sample Mendelian randomisation study
Source: Gen Psychiatr. 2022 Sep 27;35(5):e100807. doi: 10.1136/gpsych-2022-100807 (PMC9516288; doi:10.1136/gpsych-2022-100807)
Supplement: Supplementary data [file gpsych-2022-100807supp006.pdf]

Table S5. Pleiotropy test

	MR Egger regression			MR-presso				
					Estimate	SD	T-stat	p-value
Left	Egger regression intercept:	-0.0029	→	Outlier-corrected	-0.19472	0.08462	-2.3011	0.023028
	Standard error:	0.0042						
	Directionality p-value:	0.491						
Right	Egger regression intercept:	-0.0018	→	/				
	Standard error:	0.004						
	Directionality p-value:	0.661						
